# Microarray Comparison of Anterior and Posterior *Drosophila* Wing Imaginal Disc Cells Identifies Novel Wing Genes

**DOI:** 10.1534/g3.113.006569

**Published:** 2013-08-01

**Authors:** Daniel M. Ibrahim, Brian Biehs, Thomas B. Kornberg, Ansgar Klebes

**Affiliations:** * Freie Universität Berlin, Institut für Biologie, Genetik, 14195 Berlin, Germany; † Cardiovascular Research Institute, University of California, San Francisco, California; ‡ Humboldt Universität, Institut für Biologie-Zytogenetik, 10115 Berlin, Germany

**Keywords:** anteroposterior compartment border, pattern formation, expression microarray, engrailed, hedgehog

## Abstract

Signaling between cells in the anterior (A) and posterior (P) compartments directs *Drosophila* wing disc development and is dependent on expression of the homeodomain transcription factor Engrailed (En) in P cells. Downstream of *en*, posteriorly expressed Hedgehog (Hh) protein signals across the A/P border to establish a developmental organizer that directs pattern formation and growth throughout the wing primordium. Here we extend investigations of the processes downstream of *en* by using expression array analysis to compare A and P cells. A total of 102 candidate genes were identified that express differentially in the A and P compartments; four were characterized: *Stubble* (*Sb*) expression is restricted to A cells due to repression by *en*. *CG15905*, *CG16884*; *CG10200*/*hase und igel* (*hui*) are expressed in A cells downstream of Hh signaling; and RNA interference for *hui*, *Stubble*, and *CG16884* revealed that each is essential to wing development.

The subdivision of the wing imaginal disc into anterior (A) and posterior (P) compartments has several remarkable features. First, each compartment represents a defined and contiguous geographical area, and after the compartments are established in the early embryo, all descendents of the constituent cells retain the compartment identity of their ancestors, even to the adult stage (Garcia-Bellido *et al.* 1973). Second, in the wing blade primordium, the A and P compartments meet to form a remarkably straight boundary line. Third, the compartments are domains of gene expression for *engrailed* (*en*), *invected* (*inv*), and *hedgehog* (*hh*), which are expressed by all P compartment cells (Kornberg *et al.* 1985; Coleman *et al.* 1987; Tabata *et al.* 1992), and for *cubitus interruptus* (*ci*) and *patched* (*ptc*), which are expressed by all A compartment cells (Eaton and Kornberg 1990; Phillips *et al.* 1990). Other genes such as *vein* (Schnepp *et al.* 1996; Amin *et al.* 1999) and *knot/collier* (Vervoort *et al.* 1999) are expressed in a stripe that abuts the A/P compartment border’s anterior side.

Maintenance of the A/P compartment border depends upon *en*. In its absence, P cells transform into A type—they express *ci* and *ptc*, they are not confined by the A/P border and can join A cells across the border, and they make structures and patterns characteristic of the A compartment. In addition to this role as a selector gene in P cells that establishes P compartment identity and inhibits A compartment identity, *en* also creates a developmental organizer at the A/P compartment border by positively regulating *hh*. Hh made in P cells signals in a paracrine manner to A cells at the border, endowing them with organizer functionality (Basler and Struhl 1994; Tabata *et al.* 1995). Anterior cells at the border express proteins such as Decapentaplegic (Dpp) in response to Hh signaling, and the function of the organizer, which is dependent upon Dpp, regulates growth and patterning of both A and P cells (reviewed in Lawrence and Struhl 1996).

Despite our detailed understanding of these key signaling processes in wing development, many questions remain about the nature of the mechanisms that act downstream of A/P signaling. Among these are the processes that keep A and P cells separate and that define the position and shape of the border. The work described here was undertaken to identify additional genes that function at the A/P border. It sought target genes downstream of *en* and *hh* by searching for and characterizing genes with patterns of expression specific to either the A or P compartments.

We performed a global screen for genes with compartment-specific expression using expression array hybridization to compare transcript levels in A and P wing disc cells. In a previous expression microarray screen, we characterized transcripts isolated from single imaginal discs and identified and analyzed transcriptional differences between different types of discs from individual larvae (Klebes *et al.* 2002). These experiments were made possible by the application of linear RNA amplification protocols (Klebes and Kornberg 2008). In a second study, we applied this strategy to the analysis of microdissected imaginal disc cell populations in the state of transdetermination (Klebes *et al.* 2005). This investigation demonstrated that the direct microarray comparison of small cell populations that originate from the same imaginal discs is feasible. Here, we apply this strategy to a microarray comparison of sets of A and P compartment cells that had been microdissected from wing discs. This expression pattern-based approach identified 102 differentially expressed genes, of which approximately half had not been previously characterized by genetic or molecular studies. We show that *Sb* expression is downstream of En; that *CG15905*, *CG16884*, and *hui* are activated by ectopic Hh; and by using RNA interference (RNAi) knockdown, that *Sb*, *hui*, and *CG16884* are required for wing development.

## Materials and Methods

### Fly stocks

The following fly stocks were used: *w^1118^* or Oregon R for *in situ* detection experiments; *hs-flp*; *P{ry,neoFRT43D*, *y+}* and *w*; *P{w*, *FRT}43D*, *en^E^/CyO* for the generation of *en*/*inv* mutant cell clones [Df(2R)*en^E^* removes most of the *en* and *inv* transcription units (Gustavson *et al.* 1996)]; *ptc-Gal4* [a hypomorphic enhancer trap allele (Speicher *et al.* 1994)], *hh-Gal4* [an enhancer trap allele (Tanimoto *et al.* 2000)], *C765-Gal4* (Nellen *et al.* 1996), en-Gal4 (generated by Andrea Brand, FlyBase ID FBrf0098595), and UAS-GFP (Bloomington stock #4775) for *in vivo* labeling and RNAi expression; *vestigial* boundary enhancer Gal4 (*vg^BE^-Gal4*; gift from G. Schubiger) and *UAS-hh* (Ingham and Fietz 1995), and *UAS-dpp* (Bloomington stock #1486) for overexpression experiments. *UAS-RNAi* transgenic stocks were obtained from the Vienna Drosophila RNAi Center (http://stockcenter.vdrc.at), for *CG10200*: 13321, 47612, 47613, 103328; for *Sb*: 1613; for *CG15905*: 13865, 13866; for *CG16884*: 51362, 51363; from the Kyoto National Institute of Genetics Stock Center, for *CG15905*: 15905R-1; for *CG16884*: 16884R-2 and for *Sb*: 4316R-1; and from the Bloomington Drosophila Stock Center, *CG10200*: 28759.

### Immunolabeling

Imaginal discs were dissected and fixed (4% formaldehyde) following standard procedures (Sullivan *et al.* 2000). Antibodies were α-Twist (Thisse *et al.* 1988), α-Ci (Motzny and Holmgren 1995), α-Hh (Tabata and Kornberg 1994).

### RNA amplification, microarray hybridization, and data analysis

Green fluorescent protein (GFP)-labeled wing imaginal discs were microdissected under a fluorescence dissecting microscope. RNA isolation, amplification, and microarray procedures were previously described (Klebes *et al.* 2002, 2005; Klebes and Kornberg 2008). Detailed information about the microarray platform (accession number: GPL2581) and the array data from this study (accession number: GSE46601) are accessible on the Gene Expression Omnibus database, http://www.ncbi.nlm.nih.gov/geo/. In brief, hybridization probes were generated by two rounds of T7-catalyzed linear RNA amplification and labeled with Cy3 and Cy5 dyes. Reciprocally labeled probes (“dye flip”) were hybridized to custom-produced glass microarrays that contained approximately 14,000 100- to 600-bp exon sequences that were generated by polymerase chain reaction (PCR). Signal intensities were collected with a GenePix 4000A Scanner and processed with GenePix software (Molecular Devices) and global median normalized with NOMAD (http://ucsf-nomad.sourceforge.net/). We performed two kinds of data analysis. First ,we used the significance analysis of microarrays software package (SAM; Tusher *et al.* 2001) to identify 203 and 76 transcripts that are enriched in the A or P compartment, respectively (Supporting Information, Table S1). A higher stringency analysis was performed by combining the SAM statistical tools with cluster analysis (Eisen *et al.* 1998) with stringent filter settings. Expression ratios were evaluated with SAM using a Delta setting of 0.733 (9.2% false discovery rate). For the cluster analysis, we eliminated spots with a sum of median intensities <300 and without data in more than 20% of the experiments. Only those spots that show ratios greater than 1 (log2-transformed) in at least 6 of 12 experiments were considered for hierarchical clustering and generation of self organizing maps. Genes of those sub-clusters that showed predominant enrichment in one channel were further used (109 in the A group and 17 in the P group). To generate the list of 102 A- or P-enriched genes, 11 duplicate spots and 13 genes that were not identified as significant by the SAM analysis were eliminated.

### *In situ* hybridization experiments

**Figure 1:**
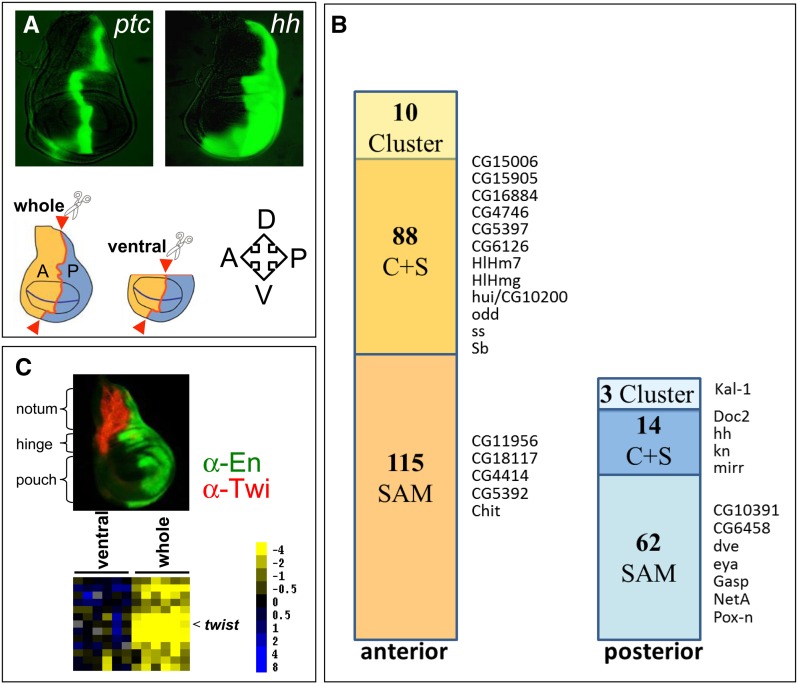
Expression array analysis identifies genes with predominant expression in A or P cells and a dorsal subcluster. ***(***A) GFP expression in the *ptc* and *hh* domains of wing imaginal discs was used to identify the compartment boundary for microdissection (genotypes: *ptc-Gal4* or *hh-Gal4* with *UAS-GFP*). Red arrowheads indicate locations of cuts that separated A and P cells of entire discs (whole) or discs without the dorsal part (ventral). The orientation of the discs is indicated: A, anterior; P, posterior; D, dorsal; V, ventral. (B) Bars indicate the numbers of transcripts identified with cluster analysis (Cluster), the significance analysis of microarrays algorithm (SAM), or both methods (S + C) to be enriched in anterior or posterior cells. The genes selected for *in situ* hybridization analysis as shown in Figure 2 are indicated. (C) The notum (dorsal) fragments identified a subcluster that includes *twist*. α-En (green) labels the P compartment and α-Twist (red) labels the adepithelial cells in the A region of the notum area. Heatmap legend shows log2-transformed ratios.

*In situ* labelings with DIG-labeled RNA probes were performed as described (Klebes *et al.* 2002). RNA probes were generated by T7 or SP6 polymerase reaction on TOPO-TA (Invitrogen Inc.) subcloned PCR products. Primer sequences are available upon request. For the *in situ* detection experiments we selected genes from both lists, that is, the SAM list of 279, and the cluster analysis-derived list of 102 genes (see Figure 1). Of the 29 selected genes, 14 were represented in both lists (Table S1), 14 represented only by SAM analysis, and one gene (*kal-1/CG6173*) was segregated into the P cluster but was not considered to be significantly enriched by the SAM analysis. Because the *in situ* pattern confirmed the expression properties for all three groups of genes, we conclude that the application of stringent filter settings eliminated many true positives. For this reason we include the complete list of significantly enriched genes in Table S1.

## Results and Discussion

### Comparison of transcript expression levels in wing disc A and P compartments

Because of the small size of the wing disc, obtaining sufficient material for microarray hybridization of defined cell populations, such as A and P cells, is challenging. We developed a method to analyze transcripts in A and P cells in single wing imaginal discs from third instar larvae. The method combines *in vivo* labeling, microdissection, linear RNA amplification, and microarray hybridization. We labeled the wing disc A/P compartment border in either of two ways, by expressing a GFP reporter transgene controlled by *ptc* or by *hh* (Figure 1A). Both reporter lines (*ptc-Gal4* and *hh-Gal4*) are weak mutant alleles for the respective genes, but neither reporter line had visible phenotypes as a heterozygote. Nevertheless, to avoid bias, we performed half of the experiments with the *ptc-Gal4* line and the other half with the *hh-Gal4* line. Wing discs have several different cell types in addition to columnar cells, including peripodial cells, associated tracheal branches, and adepithelial mesodermal cells that are present in different proportions in A and P locations. To control for the contribution and influence by transcripts from these cell types, we removed the dorsal-most part of the discs that contains most of the tracheal and adepithelial cells in half of the experiments (Figure 1A). Wing disc A and P cells were manually dissected under a fluorescence microscope and were treated separately to amplify polyadenylated RNA (Klebes and Kornberg 2008). For both the *ptc-Gal4* and *hh-Gal4* genotypes, (1) the entire A compartment was compared to the entire P compartment, and (2) the ventral A compartment was compared with the ventral P compartment; three replicates of each experiment were performed, resulting in a total of twelve. To minimize variability, the pair-wise comparisons of A and P cells were of the same imaginal disc. Microarray hybridization was performed with custom-produced glass DNA microarrays that contained approximately 14,000 short PCR-generated cDNA fragments that represent approximately 75% of the currently annotated *Drosophila* genes as well as 72 spots representing GFP gene sequences.

We applied stringent filter settings (see *Materials and Methods*) that combine cluster analysis (Eisen *et al.* 1998) and a statistical algorithm, SAM (Tusher *et al.* 2001) to the hybridization data sets. Cluster analysis identified 98 A-enriched transcripts and 17 genes with preferential expression in P cells. The SAM analysis identified 203 A and 76 P transcripts. The 102 transcripts that were common to both methods (Figure 1B, Table S1) are considered as high confidence genes with preferential A (88) or P (14) expression. The following observations support the validity of this approach. GFP expression levels in each of 12 experiments were elevated more than 12-fold in A cells in probes made from the *ptc-Gal4* line, and were elevated to similar levels in P cells for the *hh-Gal4* line (Figure S1). The average ratios of hybridization signals for the genes known to be expressed in compartment-specific patterns were also consistent with the identity of the manually isolated cells. For the 12 experiments, the average A/P ratios of the A-expressed genes *ci* and *dpp* were 9.9 and 2.2, respectively. The average P/A ratios for the P-expressed genes *en*, *hh*, and *inv* were 9.6, 11.9, and 5.1, respectively (Table 1, Table S1). Expression levels of the anteriorly expressed *ptc* gene could not be analyzed due to technical problems with the spot representing the *ptc* sequence on the microarrays.

**Table 1:** Genes with more than twofold enrichment in transcript levels in A or P compartments

Gene Name	CG#	Annotated Function (www.flybase.org)	A#/P# (ratio)
**Anterior**			
*aristaless*	CG3935	Transcription factor	20.3
*CG15611*	CG15611	Regulation of Rho protein signal transduction	11.6
*scute*	CG3827	Transcription factor	11
*cubitus interruptus*	CG2125	Transcription factor	9.9
*Drip*	CG9023	Water channel activity; cell homeostasis	7.6
*Ect3*	CG3132	Beta-galactosidase	7.2
*achaete*	CG3796	Transcription factor	7
*CG13044*	CG13044	−	6.1
*CG2663*	CG2663	Transport, vitamin E binding	5.3
*CG31705*	CG6528	−	4.9
*sister of odd and bowl*	CG6993	DNA binding	4.9
*CG13023*	CG13023	−	4.8
*CG15714*	CG15714	Protein folding	4.6
*CG13574*	CG13574	Learning or memory, olfactory learning	4.2
*CG5966*	CG5966	Lipid metabolic process, triglyceride lipase activity	4.2
*Ecdysone-dependent gene 91*	CG7539	Structural constituent of pupal cuticle	4
*E(spl) region transcript 4*	CG6099	Cell fate specification; sensory organ development	4
*drumstick*	CG10016	Nucleic acid binding; zinc ion binding	3.9
*Odorant-binding protein 56a*	CG11797	Odorant binding	3.9
*CG3244*	CG3244	Binding, C-type lectin 27kd	3.7
*CG7090*	CG7090	Oxidation-reduction process	3.7
*Imaginal disc growth factor 4*	CG1780	Imaginal disc growth factor, hydrolase activity	3.6
*pxb*	CG14874	Learning and/or memory; olfactory learning; smoothened signaling pathway	3.5
*CG5397*	CG5397	Sterol O-acyltransferase activity	3.4
*CG16884*	CG16884	−	3.3
*CG9338*	CG9338	−	3.3
*CG14598*	CG14598	−	3.2
*CG16885*	CG16885	−	3.2
*Imaginal disc growth factor 3*	CG4559	NOT chitinase	3.2
*Aldehyde dehydrogenase*	CG3752	Aldehyde dehydrogenase (NAD+)	3.1
*Antennapedia*	CG1028	Transcription factor	3.1
*CG9312*	CG9312	−	3.1
*opa*	CG1133	Transcription factor	3.1
*Actin 57B*	CG10067	Structural constituent of cytoskeleton	3
*CG10112*	CG10112	Multicellular organism reproduction, structural constituent of chitin-based cuticle	3
*CG8634*	CG8634	Structural constituent of chitin-based cuticle, Cuticular protein 65Ec	3
*Bearded*	CG3096	Calmodulin inhibitor	2.9
*CG13060*	CG13060	−	2.9
*CG18634*	CG18634	−	2.9
*phyllopod*	CG10108	Protein binding; Ras protein signal transduction; peripheral nervous system development	2.9
*CG12481*	CG12481	−	2.8
*CG15786*	CG15786	−	2.8
*CG6357*	CG6357	Cysteine-type endopeptidase activity	2.8
*CG8701*	CG8701	−	2.8
*Drop (msh)*	CG1897	Transcription factor	2.8
*CG10625*	CG10625	Structural constituent of cuticle	2.7
*CG10962*	CG10962	Oxidation-reduction process	2.7
*E(spl) region transcript g*	CG8333	Transcription factor	2.7
*Stubble*	CG4316	Serine-type endopeptidase	2.7
*CG1674*	CG1674	−	2.6
*wunen-2*	CG8805	Phosphatidate phosphatase, G-protein coupled receptor protein signaling pathway	2.6
*CG3837*	CG3837	Transmembrane receptor protein tyrosine kinase signaling pathway, protein phosphorylation	2.5
*CG5391*	CG5391	−	2.5
*CG5888*	CG5888	Transmembrane receptor activity	2.5
*CG9336*	CG9336	−	2.5
*CG10311*	CG10311	−	2.4
*CG15006*	CG15006	Structural constituent of chitin-based larval cuticle	2.4
*CG18507*	CG18507	−	2.4
*CG7924*	CG7924	−	2.4
*Pherokine 3*	CG9358	Protein serine/threonine kinase activity; carrier activity; Ras protein signal transduction	2.4
*sob*	CG3242	RNA polymerase II transcription factor	2.4
*Tetraspanin 42El*	CG12840	Receptor signaling protein activity	2.4
*CG1368*	CG1368	Structural constituent of chorion	2.3
*CG4766*	CG4766	−	2.3
*CG8483*	CG8483	−	2.3
*odd skipped*	CG3851	Transcription factor	2.3
*CG1572*	CG1572	−	2.2
*CG15785*	CG15785	−	2.2
*CG4382*	CG4382	Carboxylesterase activity	2.2
*decapentaplegic*	CG9885	Signal transducer, morphogen, growth factor	2.2
*E(spl) region transcript 7*	CG8361	Transcription factor	2.2
*CG8502*	CG8502	Structural constituent of chitin-based larval cuticle, Cuticular protein 49Ac	2.1
*blown fuse*	CG1363	Mesoderm development; myoblast fusion	2
*CG10200*	CG10200	−	2
*CG8216*	CG8216	Regulation of transcription, DNA-dependent, DNA binding	2
*CG9871*	CG9871	Translation, structural constituent of ribosome, Ribosomal protein L22-like	2
**Posterior**			P#/A# (ratio**)**
*hedgehog*	CG4637	Cysteine-type endopeptidase	11.9
*engrailed*	CG9015	Transcription factor	9.6
*invected*	CG17835	RNA polymerase II transcription factor	5.1
*mirror*	CG10601	Transcription factor, smoothened signaling pathway	3.5
*Inos*	CG11143	Enzyme, myo-inositol-1-phosphate synthase	2.9
*CG10074*	CG30837	−	2.8
*Cytochrome P450-18a1*	CG6816	Cytochrome P450	2.2

Genes are listed that show expression ratios ≥2 and that were identified by clustering and significance analysis (compare text and Table S1). Ratios were calculated with the average median intensities of the twelve arrays for A cells (A/P ratio) or P cells (P/A ratio). Ratios were rounded to one decimal place.

### The microdissection strategy identifies sets of transcripts from A, P, and dorsal wing disc cells

Among the 102 genes in the high-stringency group, 88 segregated into an A group cluster, and 17 segregated into a P cluster (Figure 1B, Table 1, and Table S1). In addition to these A and P clusters, the separate probes that were prepared from wing discs that either included or lacked the dorsal-most cells (and retained or lacked adepithelial cells) identified a subcluster associated specifically with the presence of dorsal cells. *twist* (*twi*) encodes a transcriptional activator that regulates mesodermal development and is expressed specifically in mesoderm; its transcripts were elevated in preparations containing dorsal wing disc cells (Figure 1C). The *twi*-containing subcluster also detected 12 other genes with elevated transcript levels in preparations containing dorsal cells (Table S1). This group includes several that are known or are predicted to function in either tracheal (*CG8748*; Luschnig *et al.* 2006) or muscle and mesoderm development (*CG15064*/Him, *CG6378/BM-40-SPARC*; Furlong *et al.* 2001; Swan *et al.* 2004; Liotta *et al.* 2007), or have been identified in a previously published microarray screen comparing the dorsal and ventral parts of wing imaginal discs (*eyg*, *Grip*, *CG9593*, *Him*, *CG11835*; Butler *et al.* 2003).

Neuronal cells in the wing disc arise predominantly in the primordia for the notum and anterior wing margin, the regions that will produce most of the enervated sensory organs. Both the notum and anterior wing margin primordia are predominantly in the A compartment, and genes that are known to function in neuronal development (such as *acheate* and *scute*) are therefore expected to be expressed at high levels in A cells. Furthermore, because A cells at the A/P compartment border depend upon activation of the *Notch* pathway (Casso *et al.* 2011), *Notch* pathway components might also be expressed in greater levels in A cells. The A subcluster from the array analysis did include *achaete* and *scute* as well as the *Notch* pathway components *Enhancer of spilt complex* transcripts *m7*, *m4*, and γ.

In summary, these results indicate that microdissection effectively separated A and P cells, and that the expression array hybridization identified sets of genes that are expressed in subregions of the disc. Table 1 lists the genes in the A and P clusters that had expression ratios greater than 2. We presume that putative targets of *en/*Hh regulation will be represented in the A subcluster.

### Expression patterns of candidate genes confirm hybridization array analysis

**Figure 2:**
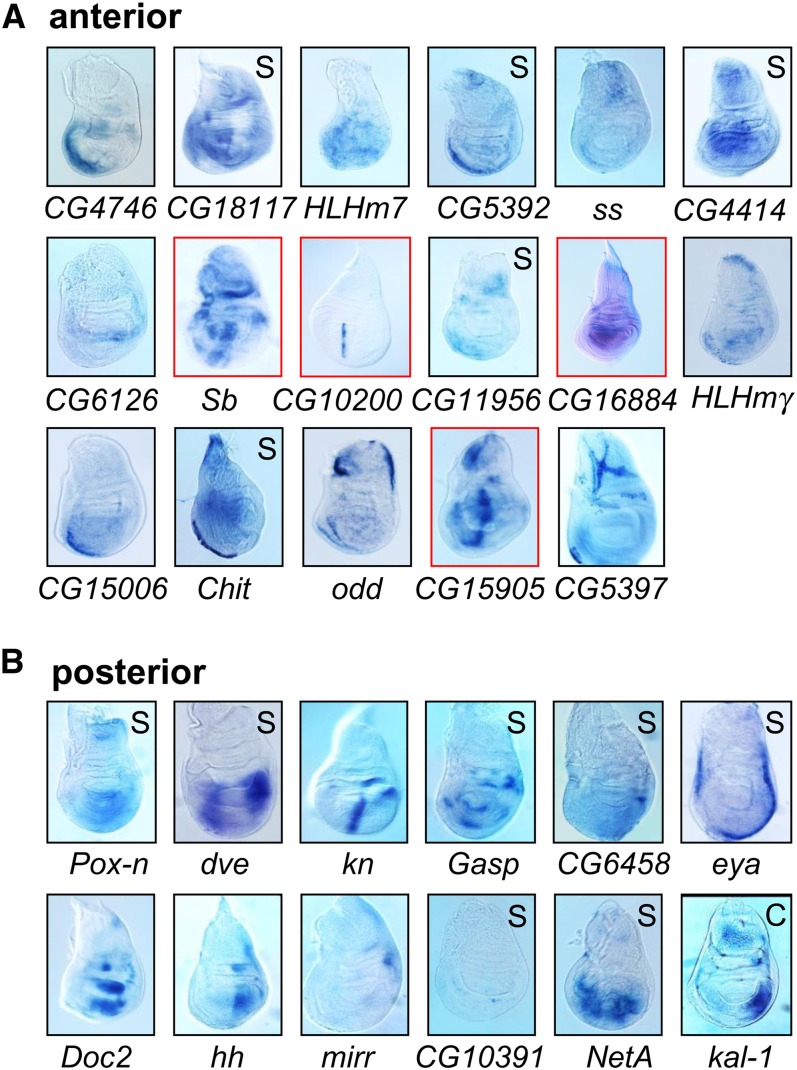
*In situ* hybridization confirms the array analysis. *In situ* hybridization was carried out for 17 genes in the A cluster and twelve genes in the P cluster. Genes were identified by cluster and SAM analysis, only by SAM (S) or only by cluster analysis (C) as indicated in Figure 1. The full gene names are provided in Table S1. Discs were dissected from wandering third instar larvae. Dorsal is up and anterior to the left in all images. Genes that were analyzed in more detail are boxed in red.

To further validate the expression array studies, we applied RNA *in situ* hybridization to whole wing discs for 29 genes. As illustrated in Figure 1B, we selected genes from each group for *in situ* analysis; 12 genes from the group of 88 that were identified by cluster and SAM analysis in the A group, five (of 115) that only SAM identified to be enriched in A cells, four of the high confidence group of 14 genes in P cells, seven (of 62) P transcripts that were identified by SAM only, and one of the three transcripts that only cluster analysis identified as P-enriched transcripts. The observed expression patterns confirmed the microarray predictions for 27 genes, revealing either predominant anterior (16 genes) or posterior (11 genes) expression (Figure 2). The only two exceptions that *in situ* hybridization did not confirm were *HLHmγ*, which in addition to the predicted A expression (by significance analysis and cluster) shows considerable expression in the P compartment, and *NetA*, which was predicted by significance analysis (but not by clustering analysis) to be predominantly in the P compartment. Note that *knot* (*kn*) is a Hh-target gene that is expressed in a stripe of A cells, but it is also expressed in a patch of cells in the P compartment (Vervoort *et al.* 1999; and Figure 2B). Because *kn* segregated with the P cluster, we assume that its expression in the P compartment was the greater influence on clustering. We conclude that the *in situ* patterns confirmed the array predictions for 27 of 29 genes. Transcripts of the A and P groups from cluster only, SAM only and the cluster and SAM overlap lists were confirmed indicating that both methods of data analysis produced list of candidate genes with a low rate of false-positive results (Table S1). The application of stringent filter settings that produced the list of 102 genes by combining cluster and significance analysis represents a high-confidence list. Based on predominantly A expression patterns, we selected four genes, *Sb*, *CG16884*, *CG15905*, and *CG10200*, for further analysis.

### Engrailed represses *Stubble* expression in posterior cells

**Figure 3:**
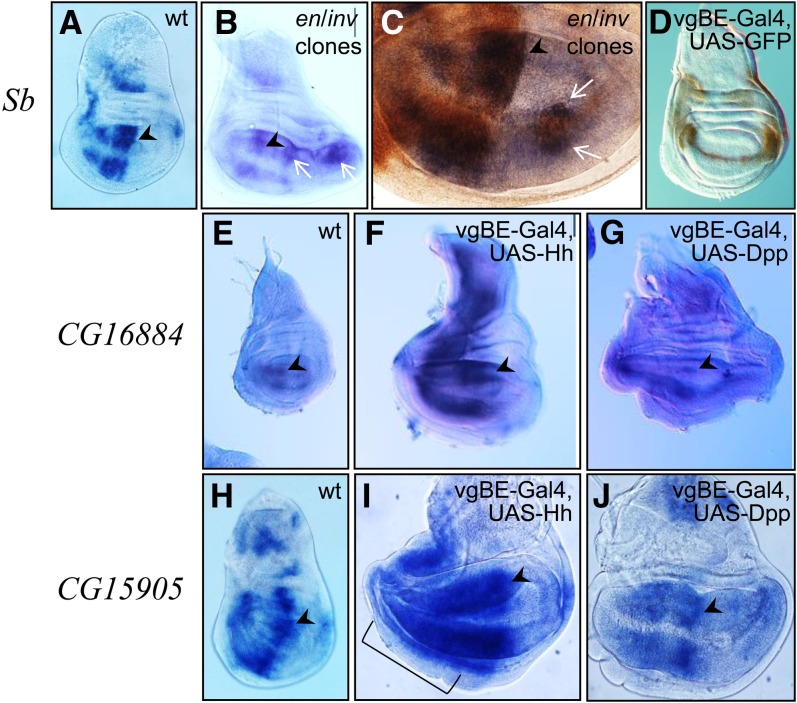
*Sb* is repressed by *en*; *CG15905* and *CG16884* respond to *hh*. *in situ* hybridization detected (A−C) *Sb* transcripts (blue) in a wild-type (wt) disc (A) and in discs with *en/inv* mutant clones (arrows, B, C). Mutant clones in the P compartment express both *Sb* (blue; *in situ* signal, arrows in B and C) and Ci (brown immunostaining in C). (D) Expression of GFP driven by the *vestigial boundary enhancer* (*vgBE-Gal4*, *UAS-GFP*) and immunostaining (HRP brown). Note the horseshoe-like pattern with a thin line along the dorsoventral compartment border. (E) *CG16884 in situ* signal in a wild-type disc is predominantly in the A compartment. (F, G) Overexpression of transgenic *hh* (F) or *dpp* (G) using *vgBE-Gal4*. Note that *hh* overexpression caused overproliferation in the A compartment and *dpp* caused overproliferation in both compartments. Signal was more intense in Hh-overexpressing discs compared with control or Dpp overexpressing discs (treated in parallel). (H−J) *in situ* hybridization detected *CG15905* expression in wild-type (H), *hh* overexpressing (I), and *dpp* overexpressing (J) discs. Note the strong *hh*-induced up-regulation of *CG15905* in the A compartment in (I), in contrast to the moderate expression levels in A cells apart from the stripe after *dpp* overexpression (J). The region with strong up-regulation is indicated by bracket in (I). Arrowheads point to the endogenous anterior stripe of expression at the A/P compartment borders; anterior, left and dorsal, up in all images. All discs are from wandering third instar larvae.

*Stubble* (*Sb*) encodes an endopeptidase that functions in cytoskeleton organization (Appel *et al.* 1993). Our microarray analysis indicated predominant anterior expression and *in situ* hybridization revealed that although many discrete areas in the wing disc expressed *Sb*, most were in the A compartment (Figure 3A). Because this expression pattern may indicate that *Sb* is repressed in P cells, we tested whether *en* has a role regulating *Sb* expression. We generated cell clones mutant for both *en* and *inv*, and in wing discs, P-cell clones lacking *en*/*inv* expressed *Sb* ectopically (Figure 3, B and C). The mutant cells also expressed *ci*, an A-specific gene that is normally repressed by *en*/*inv*. This finding indicates that En/Inv function is required to repress *Sb* in the P compartment either directly or indirectly.

### *CG16884* and *CG15905* expression is increased downstream of Hh signaling

*In situ* hybridization detected strong *CG16884* expression in the central region of the wing pouch A compartment, as well as lower level expression in some P cells and other areas of the disc (Figure 2 and Figure 3E). Expression on the anterior side of the A/P compartment border is suggestive of an activating signal from P cells, such as Hh. To test this possibility, we overexpressed a *hh* transgene in A and P cells along the dorso/ventral compartment border in the pattern of the *vestigial* boundary enhancer (vgBE; Williams *et al.* 1994, Figure 3D). This ectopic expression caused extensive over-proliferation of A cells. *In situ* detection of *CG16884* transcript revealed more intense signal in the A compartment compared to control discs (Figure 3, E and F). Because Hh up-regulates Dpp in A cells (but not in P cells), ectopic activation of *CG16884* in A cells could be a consequence of Dpp-dependent activation. We therefore tested the response of *CG16884* to ectopic expression of a *dpp* transgene by expressing Dpp under vgBE control. In these discs A and P cells over-proliferate due to the mitogenic activity of Dpp. However, *CG16884 in situ* hybridization did not reveal increased expression levels (Figure 3G).

In control discs *CG15905* is expressed in numerous patches of wing disc A cells, including a prominent broad stripe along the compartment border (Figure 3H). Its expression in the A compartment increased in response to ectopic expression of Hh (Figure 3I) but was insensitive to Dpp (Figure 3J). These findings show that *CG16884* and *CG15905* expression can be activated by ectopic Hh.

### *CG10200-hase und igel* is expressed in a dynamic pattern and responds to ectopic Hh

**Figure 4:**
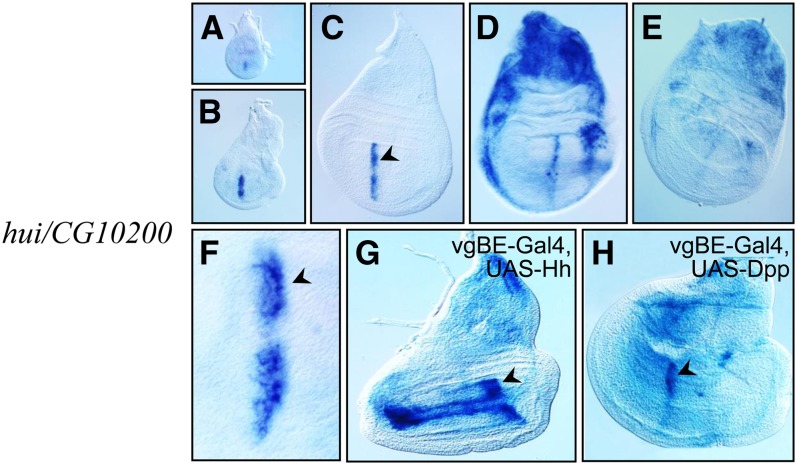
*hui/CG10200* expression changes with time and responds to ectopic *hh*. (A−E) *hui*/*CG10200* expression in wild-type young third instar (A, B), early wandering third instar (C), wandering third instar (D), and late third instar/early prepupa stage (E) discs. (F) Greater magnification view of the anterior stripe of expression from a disc comparable with (C). (G) Ectopic *hh* activates *hui/CG10200* expression in two stripes adjacent to the D/V compartment border in A cells. (H) *Dpp* overexpression does not induce ectopic *hui*. Compare the ectopic expression of *hui* to the expression domain of the *vgBE-Gal4* activator in Figure 3D.

*In situ* hybridization detected *CG10200* expression in wing discs in a narrow A-compartment stripe that abuts the A/P compartment of the wing pouch of early third instar discs (Figure 4, A−C). This stripe was four to five cells wide but was not uniformly intense. It had a gap in the area of the dorsal/ventral compartment border, and its intensity, which was greatest in the cells closest to the P compartment, decreased gradually with increasing distance from the border (Figure 4F). In late third instar discs, expression in the stripe diminished and increased both dorsally in the notum primordium and in the flanks of the disc (Figure 4D). Expression was at low levels in pre-pupal discs (Figure 4E). Monitoring the expression of *CG10200* in discs that overexpressed *hh* in the vgBE domain revealed ectopic activation in response to the Hedgehog signal (Figure 4G). *CG10200* expression was limited to two narrow stripes located at the dorsal and ventral sides of the D/V compartment border, an area that overlaps or is adjacent to the cells that expressed the *hh* transgene (compare Figure 4G with Figure 3D). In contrast to Hh overexpression, no ectopic activation of *CG10200* was observed after Dpp overexpression (Figure 4H). This result suggests that Hh, but not Dpp, regulates expression of *CG10200*. We named *CG10200 hase und igel* (*hare and hedgehog*; *hui*).

**Figure 5:**
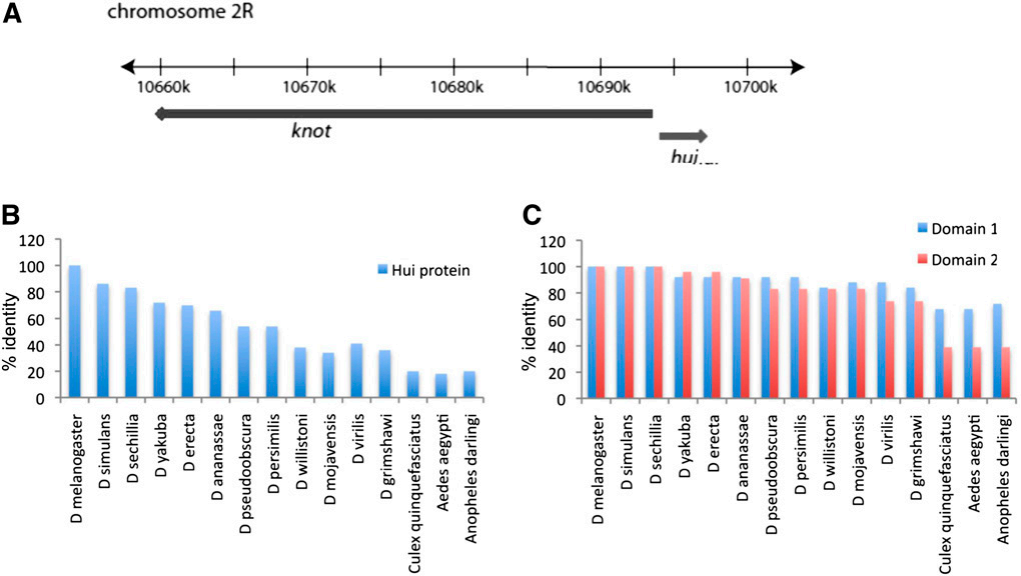
Genomic map of the *knot/hui* region and sequence conservation of the Hui protein (A) Drawing showing divergent orientations of the *kn* and *hui* genes at the indicated coordinate positions on chromosome 2R. (B) ClustalW (v1.4) comparison of the predicted *D. melanogaster* Hui sequence to Hui proteins of other Drosophilids and insects (C) and of the two most conserved regions (see Figure S3) designated arbitrarily as Domains 1 and 2. Numbers on the y-axes in B and C indicate identity in percentage.

*hui* is one of three genes identified by our analysis that is expressed in a stripe along the wing disc A/P compartment boundary. The other two genes are *CG15905* and *kn* (Figure 2). *kn* has two expression domains in third instar wing discs , a region with strong expression in the dorsal hinge primordium of the P compartment as well as the stripe in the A compartment that abuts the A/P border of the wing pouch primordium. The segregation of *kn* with the expression array posterior cluster (P/A = 1.7) indicates that the P compartment transcripts biased the cluster analysis, but the relevant and interesting issue for *hui* is that its transcription unit is immediately adjacent to *kn* (Figure 5A). Because the two genes are transcribed in opposite directions, it is possible that the 1.4-kbp intervening chromosomal region contains regulatory elements that control both genes.

The predicted Hui protein has 267 residues (Supporting Information, Table S2.) with no strong homology to known proteins or structures. It is highly conserved within the *Drosophila* genus (Figure 5B), but conservation outside the *Drosophilids* is low, and searching among genomes in the National Center for Biotechnology Information database (Basic Local Alignment Search Tool, *i.e.*, BLAST) detected homologous sequences only in other insect genomes. Several regions of high conservation were noted after ClustalW alignment of insect sequences, the two largest of which are arbitrarily denoted as domains 1 and 2 (Figure S3); these several regions of strong conservation account for the limited homology to the apparent Hui proteins in non-Drosophilid insects (Figure 5C). In addition to these regions of high conservation, the Hui protein contains a putative signal peptide sequence at the amino-terminus that could mediate targeting to the endoplasmic reticulum for secretion or targeting to other organelles (Table S2). The fact that the Hui protein has two highly conserved domains (Figure 5) suggests that it is *hui’s* protein product that is under selective pressure.

### RNAi knockdown indicates a requirement for *Sb*, *CG16884*, and *hui* during wing development

#### RNAi knockdown specificity:

To investigate how *Sb*, *CG16884*, *CG15905*, and *hui* function in wing development, we examined hypomorphic conditions for each gene by using RNAi to knock down expression in different regions of wing discs. Three lines expressing Gal4 were used: *ptc-Gal4*, which expresses in the A compartment with greatest expression levels in a stripe along the compartment border (Figure 1A); *en-Gal4*, which expresses in the P compartment (Figure 1C); and *C765-Gal4*, which expresses in most wing disc cells. We first monitored the specificity of the knockdown effects. The only extant mutant alleles of these genes are *Sb* mutants that are characterized by short, stubby bristles. By expressing RNAi directed against *Sb* RNA with *C765-Gal4* throughout the wing disc, we selectively phenocopied this short, thick bristles phenotype in the notum and scutellum (not shown). This result is consistent with a previous report, which describes *Sb*-directed RNAi driven by *Act5c-Gal4* (Dietzl *et al.* 2007). Importantly, head bristles, which are abnormal in *Sb* mutants, were normal in *C765-Gal4 Sb*-RNAi flies, indicative of the specific spatial targeting of the knock-down to the wing disc.

To estimate the efficiency of RNAi knock-down, we analyzed *hui* transcript levels by semiquantitative reverse transcription PCR. Transcript levels for *hui* were reduced relative to our reference *Actin 42A* in wing discs that activated expression of the RNAi construct in most cells of the wing imaginal disc (Figure S2). Three independent transgenic insertion lines for this construct showed comparable phenotypic effects (Please see *RNAi knockdowns of Sb, CG16884, and hui/CG10200 cause wing malformations*), indicating that the phenotypes were not caused by position effect of the transgenic insertions. However, because several putative off-targets were predicted for this RNAi construct (http://stockcenter.vdrc.at), additional tests were made of the specificity of the *hui* knockdown. First, we used three transgenic lines that carry different *hui* RNAi constructs and these independent transgenic lines produced comparable RNAi phenotypes (see next section). Second, we analyzed the transcript levels of one of the predicted off-targets, the *BRDW3/CG31132* gene. We selected this gene because *BRDW3/CG31132* is the most likely candidate to show effects on wing development, particularly on vein formation. RT-PCR analysis of *BRDW3/CG31132* revealed no obvious reduction in levels of this transcript (Figure S2). We conclude that the effects of the *hui* RNAi-mediated knockdown are likely to be specific for the *hui* function.

#### RNAi knockdowns of Sb, CG16884, and hui/CG10200 cause wing malformations:

**Figure 6:**
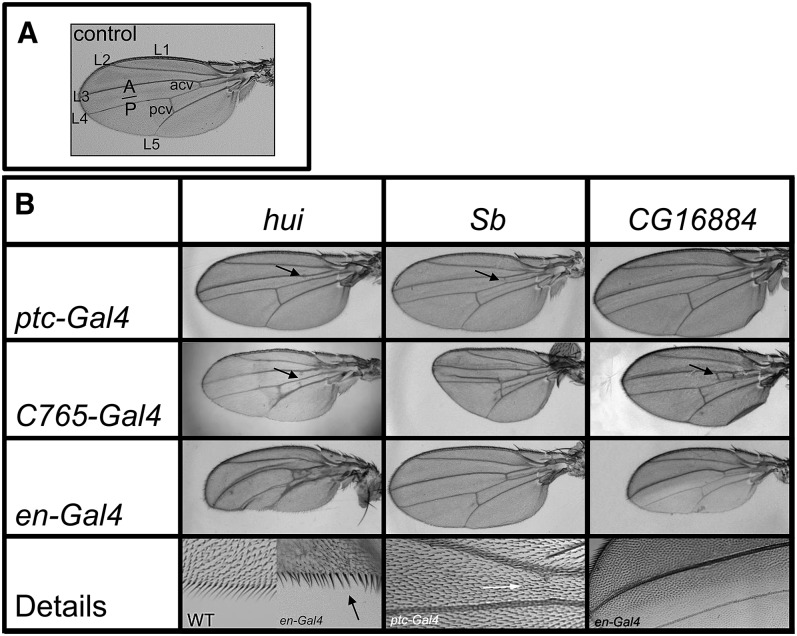
RNAi knockdown reveals requirement for *Sb*, *CG16884*, and *hui* in wing development (A) Wild-type wing, with longitudinal veins L1-5, anterior (acv) and posterior crossveins (pcv) and the A/P compartment border indicated. (B) RNAi constructs directed against *hui*, *Sb*, and *CG16884* (columns) were expressed along the A/P border (*ptc-Gal4*, top row), throughout the disc (*C765-Gal4*, middle row), or in the P compartment (*en-Gal4*, third row). *hui*-RNAi driven by *ptc-Gal4* resulted in stubby wings with narrowed L3-L4 intervein region and missing acv (indicative of Hh signaling deficits, arrow). When driven by *C765-Gal4* and *en-Gal4*, these wings have abnormal shape and venation. Phasing of the bristles at the posterior wing margin was disturbed following activation with *en-Gal4* (arrow in detail view showing a comparison to a wild-type margin). Expression of *Sb* RNAi under *ptc-Gal4* reduced or eliminated the acv (arrow in top left and detail view bottom row, compare to control in (A). Expression of *Sb* RNAi under *C765-Gal4* reduced the size of both A and P compartments and caused some ectopic vein formation. *Sb* RNAi in P cells (*en-Gal4*) caused no apparent effect. *CG16884*-RNAi in the *ptc* domain caused narrowing of the L3-L4 intervein region. Knock-down with C765-*Gal4* resulted in ectopic vein formation (arrow); RNAi in P cells caused size reduction of the P compartment and a fragile and less pigmented appearance of the cuticle (detail view). All images except for the detail views are to scale.

Knockdown of *Sb* expression reduced the size of the wing and caused vein malformations (Figure 6). RNAi expression in the *ptc* domain narrowed the L3-L4 intervein region and reduced the anterior crossvein (acv). Expression driven by *C765-Gal4* reduced the size of both A and P compartments and appeared to primarily affect proximo-distal growth. Wings that developed in the *C765-Gal4 Sb*-RNAi genotype were 32% shorter than controls (*t*-test *P*-value <2.2E-8). In some cases wings were significantly smaller than the presented example and many were crumpled (not shown). In addition, vein bifurcations and ectopic veins, including a stretch positioned anterior to L4, were observed frequently. Induction of RNAi exclusively in the P compartment (*en*-Gal4 *Sb*-RNAi) had only mild effects. The overall wing size was not affected, and bifurcations of the posterior cross vein (pcv) that occurred were rare.

Whereas previous studies detected *Sb* only during metamorphosis (Appel *et al.* 1993), our results show that it is expressed earlier, in third instar discs. The importance of larval disc expression may be related to a more pleiotropic role for *Sb*. Various allelic combinations, such as *Sb*/*stubbloid* and *Sb^1^/Sb^63b^*, affect both leg and wing morphogenesis (Beaton *et al.* 1988). Interestingly, *Sb^1^/Sb^63b^*, wings are reduced in size and have an ectopic vein anterior to L4, abnormalities that are nearly indistinguishable from wings after *C765-Gal4*-mediated *Sb* knockdown (Figure 6). Consistent with its predominant anterior expression this function of *Sb* in wing development appears to be required in A cells, because *Sb* RNAi expression in the P compartment (with *en-Gal4*) revealed no or only very mild phenotypic defects. Additionally, the absence of an anterior crossvein in wings produced after *ptc-Gal4*−mediated knockdown suggests a role downstream of Hh in the L3-4 intervein region. It is interesting to note that in flies subjected to *C765-Gal4*-mediated *Sb* knock-down, both A- and P-wing compartments are abnormally small. We speculate that compensatory regulation between the two compartments may account for this effect.

Knockdown of *CG16884* in the *ptc* pattern caused narrowing of the L3-L4 intervein region similar to the *Sb* and *hui* (see next section: *hui RNAi and knot loss-of-function cause similar wing defects*) RNAi phenotypes. In contrast to the other two genes, however, the acv was not ablated (Figure 6), although in a few wings the acv had small gaps (not shown). *C756-Gal4*−mediated RNAi resulted in slightly smaller wings with some ectopic vein formation, especially in the position of the anterior and posterior crossveins. A reduction in size was also observed after *en-Gal4* activation. Similar to *hui* (see next section), the reduction affected the P compartment causing a posterior curvature of the wing. In addition to the growth disadvantage, *en-Gal4*−mediated RNAi resulted in defects in cuticle formation—the cuticle of the entire P compartment appeared fragile and less pigmented.

Expression of the *CG15905* RNAi construct did not produce obvious defects with any Gal4 line that we tested (not shown). We do not know whether the absence of a morphological phenotype is a consequence of lack of requirement, unidentified genes with redundant function or insufficient knockdown.

#### hui RNAi and knot loss-of-function cause similar wing defects:

Phenotypes produced by all three of the *hui*-RNAi constructs we tested were indistinguishable; Figure 6 shows representative examples with characteristic defects that were observed. *hui* knockdown in the region between wing veins 3 and 4 with *ptc-Gal4* ablated the acv and narrowed the L3-L4 intervein region, and in some wings, gaps in L3 were also observed (not shown). Expression throughout the wing using *C765-Gal4* also caused L3-L4 malformation and occasional partial ablation of the acv (not shown). These phenotypes are characteristic of reduced Hh signaling (for example, see Casso *et al.* 2008), and of *kn* mutant phenotypes (Nestoras *et al.* 1997; Vervoort *et al.* 1999). Thus, *hui* and *kn*, whose expression at the A/P boundary is similarly responsive to Hh signaling, are both required for the intervein L3-L4 region.

Additional phenotypes induced by *hui* RNAi that are not observed for *kn* include a significant reduction in size of the wing and irregularly spaced bristles along the wing posterior margin (Figure 6). The small wing phenotype was observed after knockdown of *hui* throughout the wing disc (*C765-Gal4*; the entire wing was small) or specifically in the P compartment (*en-Gal4*; only the P compartment was small). In *en-Gal4* wings, the L3-L4 intervein region was not reduced and the acv was present. Because the *en-Gal4* induced phenotype did not affect the size or appearance of the L3-L4 intervein region, which is the most sensitive area of the wing to changes in Hh signaling, these effects of *hui* knockdown in the P compartment appear to be autonomous to the cells with reduced *hui* expression. This reasoning leads us to suggest that the targets of *hui* knock-down that are affected by *en-Gal4* and *C765-Gal4* knock-down are the *hui*-expressing cells along the flanks of the disc rather than the A stripe. If true, this would suggest that in addition to the *kn*-like function in A cells the growth of the wing primordium is dependent on *hui* expression in cells of the hinge primordium and/or the flanks of the wing pouch.

#### Anterior and posterior cells differ in expression of at least 102 genes:

In 1975, Garcia-Bellido proposed the term “selector gene” to designate the key regulatory genes that control the growth and differentiation of the groups of cells that populate developmental compartments (Garcia-Bellido 1975). His proposal was based on genetic studies of homeotic genes and of *en*, which had been shown to be specifically required in P-compartment cells of the wing disc (Garcia-Bellido *et al.* 1973; Morata and Lawrence 1975). Subsequent molecular studies have fully validated the selector gene hypothesis: the homeotic genes and *en* have expression patterns that correlate precisely with the cells that require their functions.

In the third instar wing disc, *en* is expressed in all P compartment cells (Kornberg *et al.*1985). Three other genes that are known to be expressed in all cells of either the A or P compartments are *ci* (A) and *inv* and *hh* (P). These genes were first isolated by either positional cloning based on the phenotype of insertional mutants (*e.g.*, *ci*, Orenic *et al.* 1990; *hh*, Mohler and Vani 1992), by linkage and coregulation with *en* (Coleman *et al.* 1987), or based on their compartment-specific expression patterns that were revealed in enhancer trap lines (*e.g.*, *ci*, Eaton and Kornberg 1990; and *hh*, Lee *et al.* 1992; Tabata *et al.* 1992). The compartment-specific expression of these four genes led in the ensuing years to numerous enhancer trap screens in search of other genes that express in a compartment-specific patterns, but these efforts identified no other genes that are expressed specifically in all A or P cells (T. Kornberg, unpublished data). It has not been apparent whether these negative results were due to the inadequacy of the insertional-based approaches used to screen for expression patterns or to the absence of other such genes.

The expression array screen described here was based on manual dissection and isolation of small numbers of cells from single imaginal discs. The cells were identified by patterns of GFP expression regulated either by *ptc-Gal4* or *hh-Gal4*, and our results showed that the levels of GFP transcripts in these isolated cells correlated strongly with the domains that express GFP. The expression array analysis monitored approximately 75% of the currently annotated *Drosophila* genes and identified a list of 102 genes with preferential A or P expression. The three genes with the greatest P/A expression ratio were *en*, *hh*, and *inv*. The identification of the same set of genes by two dissimilar genome-wide scans (*e.g.*, expression array hybridization and enhancer trap screening) suggests that *en*, *hh* and *ci* may be unique among *Drosophila* genes in their compartment-specific expression. Thus, whereas *en*, *hh*, and *ci* had the most robust differences in expression levels, the other genes in our list produced more moderate differences in signal intensities (Table 1 and Table S1). Most of these genes appear to be expressed by subsets of cells in both compartments with only moderate enrichment in one or the other (Figure 2). Nevertheless, several expression patterns are suggestive of regulation downstream of *en* or *hh*, and we analyzed four of these genes: *Sb*, *CG16884*, *CG15905*, and *CG10200*/*hui*, of which three are involved in wing development. The data we present indicate that *Sb* is a regulatory target of *en*/*inv* and that *hui*, *CG16884*, and *CG15905* are inducible by the *en*/*inv*-regulated gene *hh*. It is interesting to note that the Sb protein is thought to be a transmembrane serine protease that could process as yet unidentified signaling proteins (Bayer *et al.* 2003), and that *hui*, *CG15905*, and *CG16884* encode short proteins with amino-terminal putative endoplasmic reticulum signal peptides Table S2. These observations raise the possibility that all three proteins could be secreted or could process secreted factors that act on cells beyond their expression domains.

## Supplementary Material

Supporting Information
