# Supplementary material for: Microarray Comparison of Anterior and Posterior Drosophila Wing Imaginal Disc Cells Identifies Novel Wing Genes
Source: G3 (Bethesda). 2013 Aug 1;3(8):1353–62. doi: 10.1534/g3.113.006569 (PMC3737175; doi:10.1534/g3.113.006569)
Supplement: Supporting Information [file supp_3_8_1353__index.html]

Microarray Comparison of Anterior and Posterior Drosophila Wing Imaginal Disc Cells Identifies Novel Wing Genes — Supporting Information 

# Microarray Comparison of Anterior and Posterior *Drosophila* Wing Imaginal Disc Cells Identifies Novel Wing Genes

## Supporting Information for Ibrahim *et al.*, 2013

**Files in this Data Supplement:**

- Supporting Information - Figures S1-S3 and Table S1 (PDF, 1 MB)
- Figure S1 - Quality control of A/P microarray comparison (PDF, 82 KB)
- Figure S2 - Hui protein alignment (PDF, 98 KB)
- Figure S3 - RT-PCR quantification of *hui* expression levels after RNAi knockdown (PDF, 1 MB)
- Table S2 - Protein sequences and prediction of signal peptides (PDF, 160 KB)
- Table S1 - List of genes with differential A and P expression (.xls, 625 KB)
