# Supplementary material for: Microarray Comparison of Anterior and Posterior Drosophila Wing Imaginal Disc Cells Identifies Novel Wing Genes
Source: G3 (Bethesda). 2013 Aug 1;3(8):1353–62. doi: 10.1534/g3.113.006569 (PMC3737175; doi:10.1534/g3.113.006569)
Supplement: Supporting Information [file supp_g3.113.006569_006569SI.pdf]

G3 Genes | Genomes | Genetics

Microarray comparison of anterior and posterior *Drosophila* wing imaginal disc cells identifies novel wing genes

Daniel M. Ibrahim[*1], Brian Biehs[§2], Thomas B. Kornberg[§], and Ansgar Klebes[*$3]

[*] Freie Universität Berlin, Institut für Biologie, Genetik, 14195 Berlin, Germany
[§] Cardiovascular Research Institute, University of California, San Francisco, USA
[1] current address: Max Planck Institute for Molecular Genetics, 14195 Berlin – Germany
[2] current address: Department of Molecular Biology at Genentech, Inc., South San Francisco, California, USA.
[3] current address: Humboldt Universität Berlin, Institut für Biologie, Zytogenetik, 10116 Berlin, Germany

DOI: 10.1534/g3.113.006569

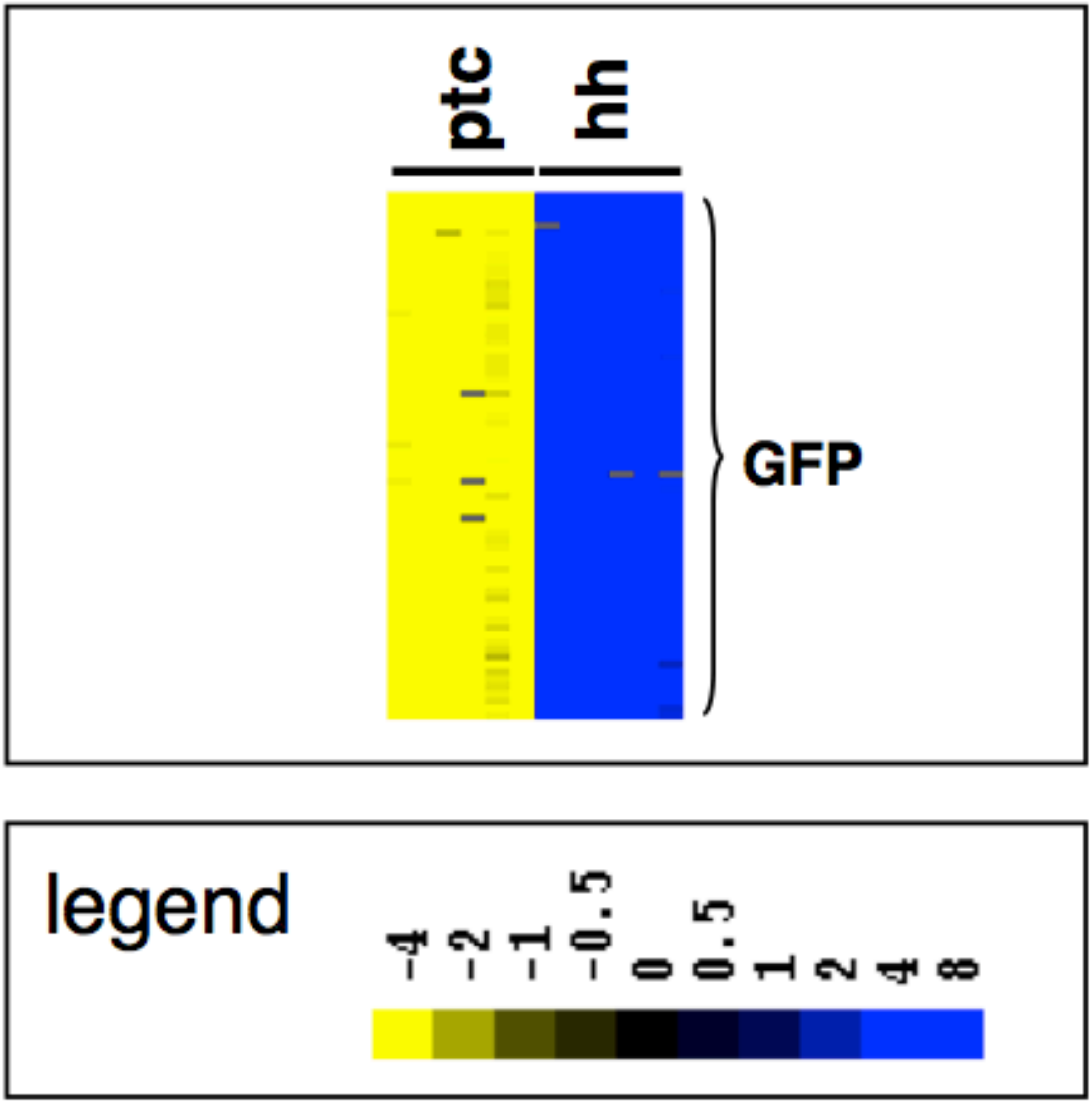

Figure S1 Quality control of A/P microarray comparison. Heat-map of GFP expression in 72 replicate GFP spots. *ptc-Gal4* activated GFP expression resulted in anterior hybridization to GFP (yellow); and *hh-Gal4* activated GFP expression resulted in posterior hybridization to GFP (blue) (compare to Figure 1A). The twelve individual array hybridizations are indicated in the columns and the 72 replica GFP spots in the rows. Legend of the color-coded expression ratios in log2-transformed values.

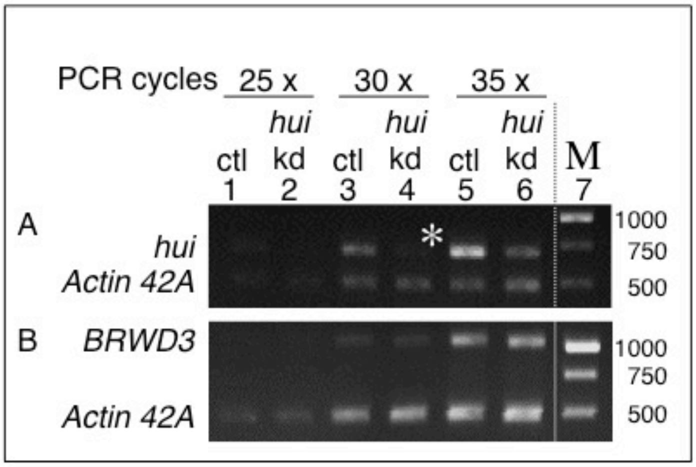

Figure S2 RT-PCR quantification of *hui* expression levels after RNAi knockdown. (A, B) RNA was isolated with RNA purification Mini kit (Zymo Inc.) from wing discs of control larvae (ctl, *white*^{1118}) and larvae that expressed the transgenic *hui* RNAi construct under control of the *C765-Gal4* (hui kd). Duplex RT-PCR for *hui* and *Actin 42A* (as reference) or *BRWD3/CG31132* and *Actin 42A* was performed with the OneStep RT-PCR kit (Qiagen). Primers were: GTCGTTTTGGAACTTTTCACGCGTTC (hui-forward), GTTCTATTTGGATGAGTAAGTGGATTCGG (hui-reverse), CTGGGTCTGCCGATCACCATGTG (BRWD3-forward), CATTTACATTAACCGACTGCCGATTGGC (BRWD3-reverse) GATGGCAACATACATGGCCG (Actin 42A-forward), GTGTGCAGCGGATAACTAG (Actin 42A-reverse). The annealing temperature was 50° C and 10 μl aliquots were removed after 25, 30, and 35 PCR cycles. (upper) Note that *hui* transcript levels (*) are reduced in the RNAi discs relative to *Actin 42A* levels. (lower) Quantification of the predicted off-target transcript *BRWD3* in control discs, showing no reduction after *hui* knock-down. M - marker. Numbers indicate size (base pairs).

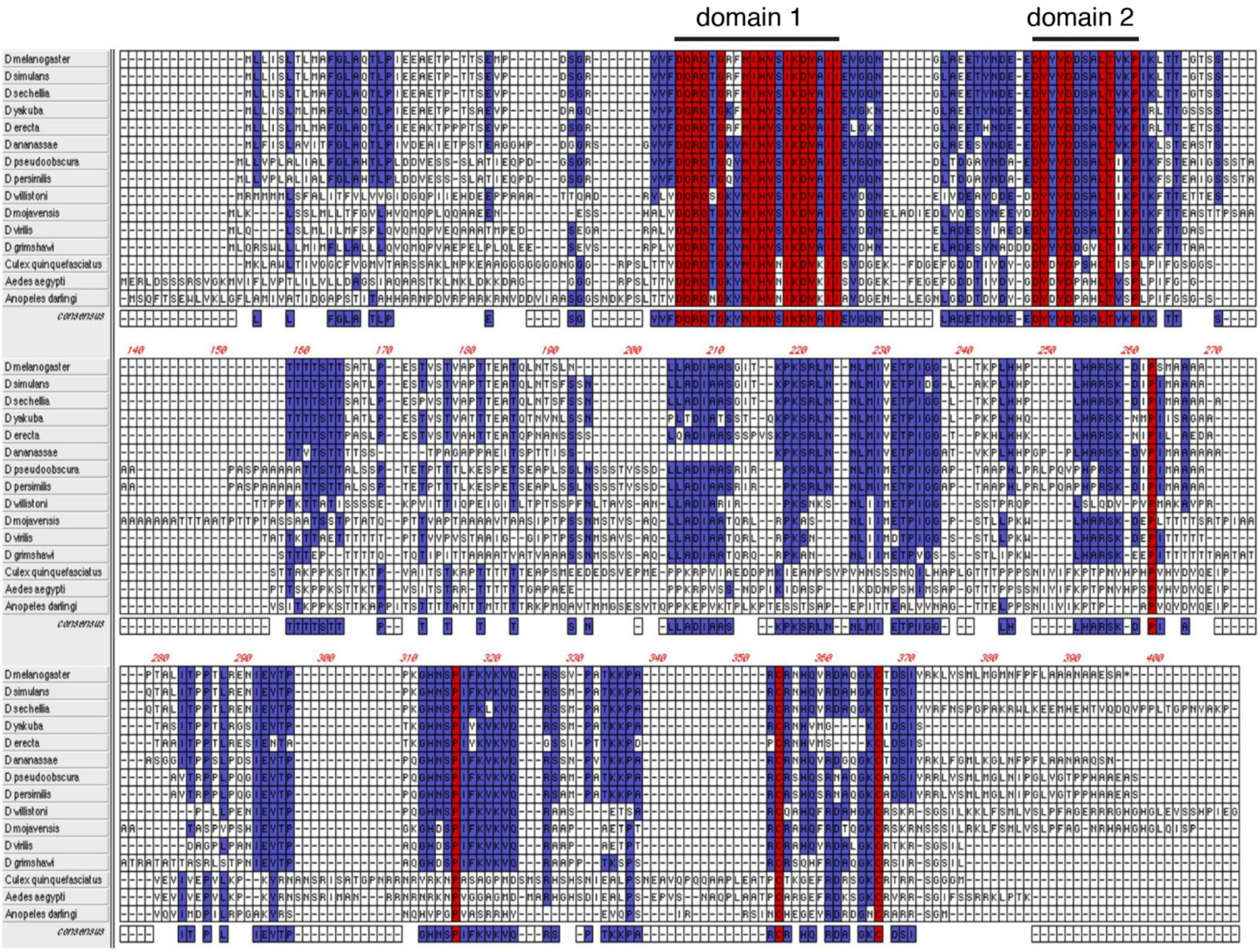

Figure S3 Hui protein alignment. The putative Hui protein of *D. melanogaster* was aligned with homologs from eleven other *Drosophila* and three other insect species. Domains of highest conservations indicated; blue indicates strong conservation; red, complete identity. The positions of the arbitrarily determined Domains 1 and 2 are indicated.

Table S1 List of genes with differential A and P expression. Datasheet that includes A. the list of A and P genes that were identified with SAM, B. the list of 102 A and P genes that were identified with a combination of cluster analysis and SAM, C. the list of the notum sub-cluster, and D. the list of 29 genes that were analyzed by *in situ* detection. For a description of data analysis see Material and Methods. The original microarray data is available at: http://www.ncbi.nlm.nih.gov/projects/geo/index.cgi; accession number GSE46601.

Table S1 is available for download at http://www.g3journal.org/lookup/suppl/doi:10.1534/g3.113.006569/-/DC1.

Table S2 Protein sequences and prediction of signal peptides. The predictions for signal peptides were generated with the SignalP 4.1 software (http://www.cbs.dtu.dk/services/SignalP/) from the Center for Biological Sequence Analysis of the Technical University of Denmark Lyngby.

```
SignalP 4.0: discriminating signal peptides from transmembrane regions
Petersen TN., Brunak S., von Heijne G. & Nielsen H.
Nature Methods, 8:785-786, 2011
```

CG10200 (*hui*) predicted Protein 1

```
MKYAVIAIALFAITTASASSAGQFLPRAFFTLDSEGHQSNVHPVNAHLLR
RLRRQSSSSSSSSSSSSSSSGGNVFTYASHSVQNADGSGHSGSSYTQQAAA
PVLGGVSFDERFGESSLTGGDNYYPTYNGYTSAASISSNSGVGSTGSYQH
ISGVGPIPATQIQQTVAVVDASGNQKVTNIQGATDSNGVLNIKKTESTYS
SK
```

```
# SignalP-4.1 euk predictions
```
>Sequence

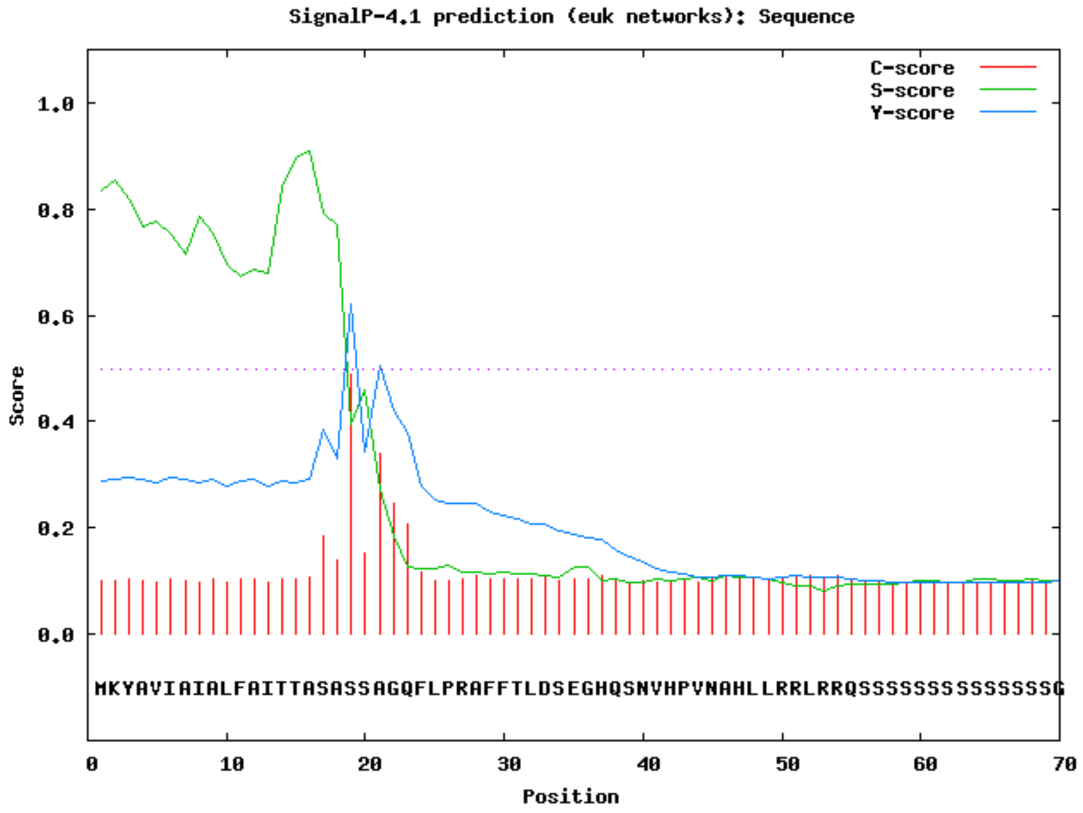

# Measure	Position	Value	Cutoff	signal peptide?
max. C	19	0.490		
max. Y	19	0.621		

```
  max. S    16       0.910

  mean S     1-18    0.778

       D     1-18    0.706   0.340   YES

Name=Sequence  SP='YES' Cleavage site between pos. 18 and 19: ASA-SS D=0.706
D-cutoff=0.340 Networks=SignalP-noTM
```

[data]

[gnuplot script]

Signal peptides: 1
[processed fasta entries]
[gff file of processed entries]

CG10200 (*hui*) predicted Protein 2

```
MKYAVIAIALFAITTASASSAGQFLPRAFFTLDSEGHQSNVHPVNAHLLR
RLRRQSSSSSSSSSSSSSSGGNVFTYASHSVQNADGSGHSGSSYTQQAAA
PVLGGVSFDERFGESSLTGGDNYYPTYNGYTSAASISSNSGVGSTGSYQH
ISGVGPIPATQIQQTVAVVDASGNQKVTNIQGATDSNGVLNIKKTESTYS
SK
```

```
# SignalP-4.1 euk predictions
```
>Sequence

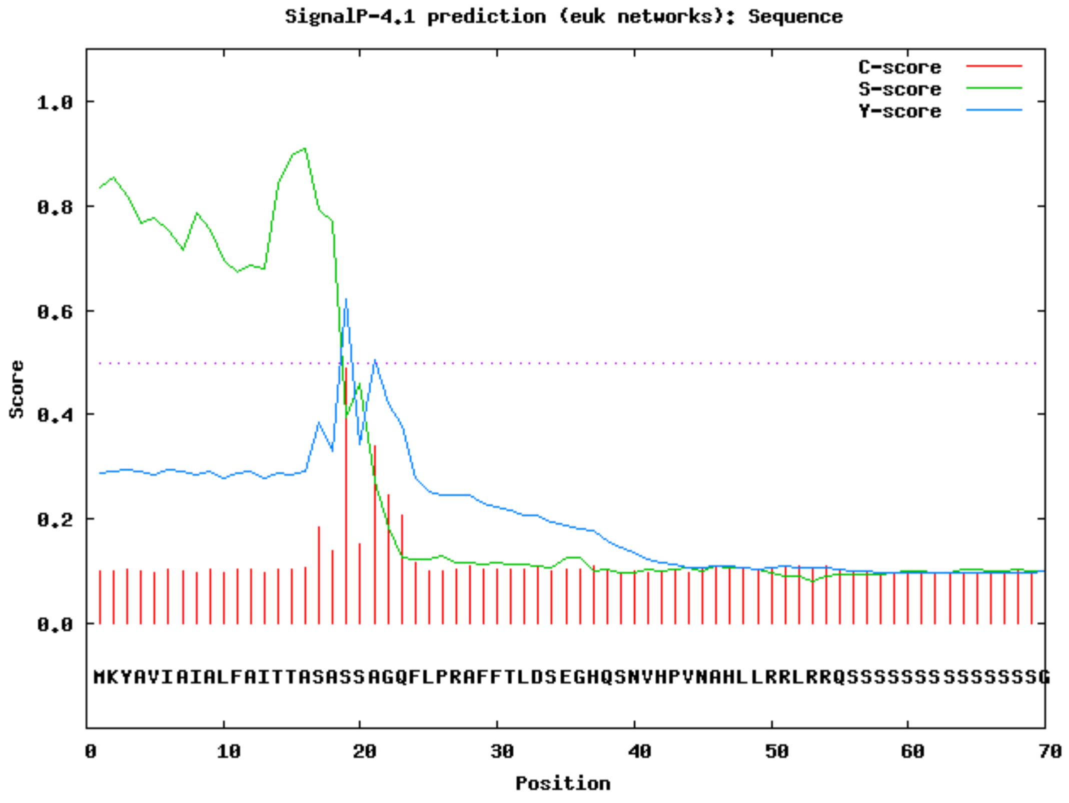

# Measure	Position	Value	Cutoff	signal peptide?
max. C	19	0.490		
max. Y	19	0.621		
max. S	16	0.910		
mean S	1-18	0.778		
D	1-18	0.706	0.340	YES

Name=Sequence SP='YES' Cleavage site between pos. 18 and 19: ASA-SS D=0.706 D-cutoff=0.340 Networks=SignalP-noTM

data

gnuplot script

Signal peptides: 1
processed fasta entries
gff file of processed entries

CG16884 predicted Protein

MKVFICLAALLVASACASKTEGEKVPLEKKLDKRGLLDLGYGYGHAGLDT
GYLGHGSISGHGSYGHGYGLTGYSAPAAVAVGHSGPAIAVGHTAPAVAVH
HAPAPYVISKQADVHKTITITKGIPVPVHVDRPYPVVHEKRVPVEVKVPV
PQPYEVIRKVPVTVKEYVKVPVPVPQPYEVIRHEKVPVHVPVDRPVPVEV
PRPYPVPVAKPYPVYVEKAVNVQVPVHVDRPYPVYVKVPVVSHSVVKHAP
TVAVSSYPVSAIGHDATVYSDHHGYHK

SignalP-4.1 euk predictions

>Sequence

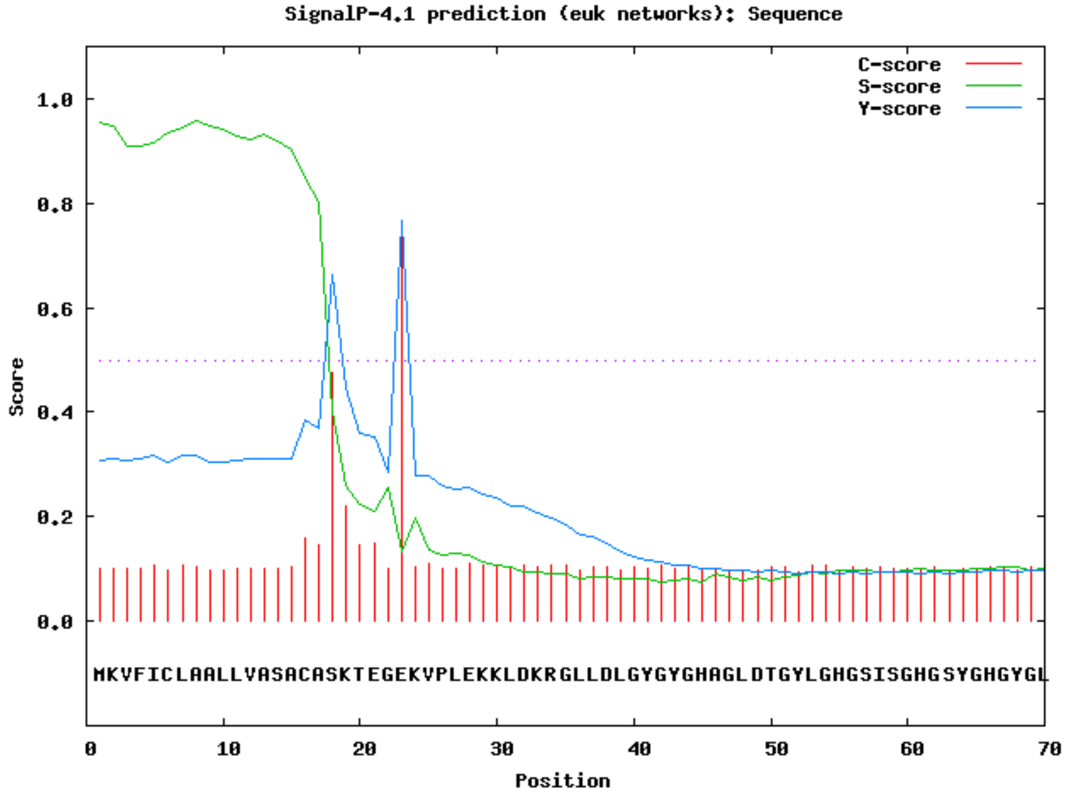

# Measure	Position	Value	Cutoff	signal peptide?
max. C	23	0.762		
max. Y	23	0.767		
max. S	8	0.958		
mean S	1-22	0.772		

D 1-22 0.770 0.340 YES

Name=Sequence SP='YES' Cleavage site between pos. 22 and 23: TEG-EK D=0.770 D-cutoff=0.340 Networks=SignalP-noTM

data

gnuplot script

Signal peptides: 1
processed fasta entries
gff file of processed entries

CG15905 predicted Protein

MLLISLTLMAFGLAQTLPIEEAETPTTSEMPDSGRVVFDQRQTGRFNIHV
SIKDVAIIEVGQNGLAEETYNDEEDYYYDDSALTVKPIKLTTGTSSTTTT
STTSATLPESTVSTVAPTTEATQLNTSLNLLADIAASGITKPKSRLNNLM
IVETPIGGLTKPLHHPLHARSKDIPSMAAAAPTALITPPTLRENIEYTPP
KGHNSPIFKVKVQRSSVPATKKPARCRNHQVRDAQGKCTDSIYRKLYSML
MGMNFPFLAAANAAESA

SignalP-4.1 euk predictions
>Sequence

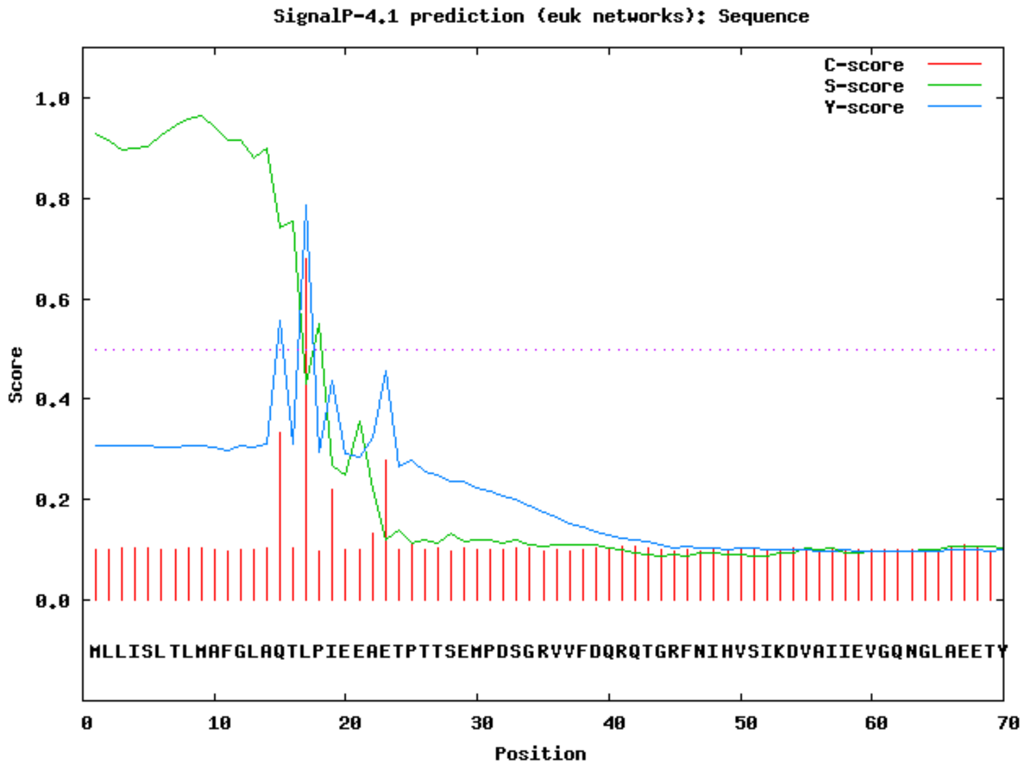

```
# Measure  Position  Value    Cutoff   signal peptide?

  max. C    17       0.680

  max. Y    17       0.785

  max. S     9       0.963

  mean S     1-16    0.899

       D     1-16    0.846   0.340   YES

Name=Sequence  SP='YES' Cleavage site between pos. 16 and 17: AQT-LP D=0.846
D-cutoff=0.340 Networks=SignalP-noTM

# data

# gnuplot script
```

```
Signal peptides: 1
# processed fasta entries
# gff file of processed entries
```
