# Supplementary material for: Microarray Comparison of Anterior and Posterior Drosophila Wing Imaginal Disc Cells Identifies Novel Wing Genes
Source: G3 (Bethesda). 2013 Aug 1;3(8):1353–62. doi: 10.1534/g3.113.006569 (PMC3737175; doi:10.1534/g3.113.006569)
Supplement: Supporting Information [file supp_g3.113.006569_FigureS2.pdf]

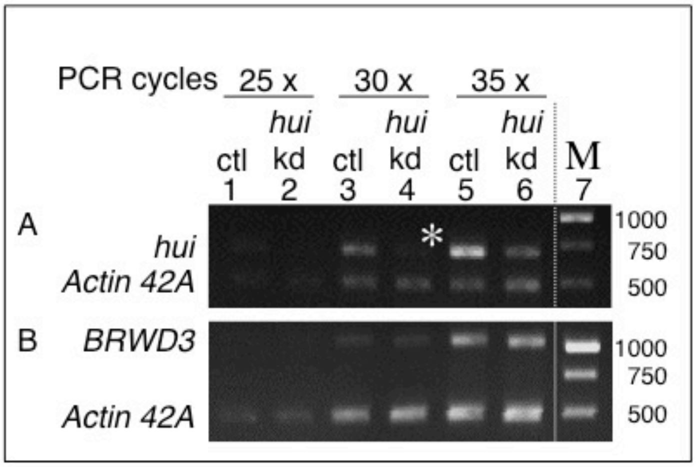

Figure S2 RT-PCR quantification of *hui* expression levels after RNAi knockdown. (A, B) RNA was isolated with RNA purification Mini kit (Zymo Inc.) from wing discs of control larvae (ctl, *white*^{1118}) and larvae that expressed the transgenic *hui* RNAi construct under control of the *C765-Gal4* (hui kd). Duplex RT-PCR for *hui* and *Actin 42A* (as reference) or *BRWD3/CG31132* and *Actin 42A* was performed with the OneStep RT-PCR kit (Qiagen). Primers were: GTCGTTTTGGAACTTTTCACGCGTTC (hui-forward), GTTCTATTTGGATGAGTAAGTGGATTCGG (hui-reverse), CTGGGTCTGCCGATCACCATGTG (BRWD3-forward), CATTTACATTAACCGACTGCCGATTGGC (BRWD3-reverse) GATGGCAACATACATGGCCG (Actin 42A-forward), GTGTGCAGCGGATAACTAG (Actin 42A-reverse). The annealing temperature was 50° C and 10 μl aliquots were removed after 25, 30, and 35 PCR cycles. (upper) Note that *hui* transcript levels (*) are reduced in the RNAi discs relative to *Actin 42A* levels. (lower) Quantification of the predicted off-target transcript *BRWD3* in control discs, showing no reduction after *hui* knock-down. M - marker. Numbers indicate size (base pairs).
