# Supplementary material for: Microarray Comparison of Anterior and Posterior Drosophila Wing Imaginal Disc Cells Identifies Novel Wing Genes
Source: G3 (Bethesda). 2013 Aug 1;3(8):1353–62. doi: 10.1534/g3.113.006569 (PMC3737175; doi:10.1534/g3.113.006569)
Supplement: Supporting Information [file supp_g3.113.006569_FigureS3.pdf]

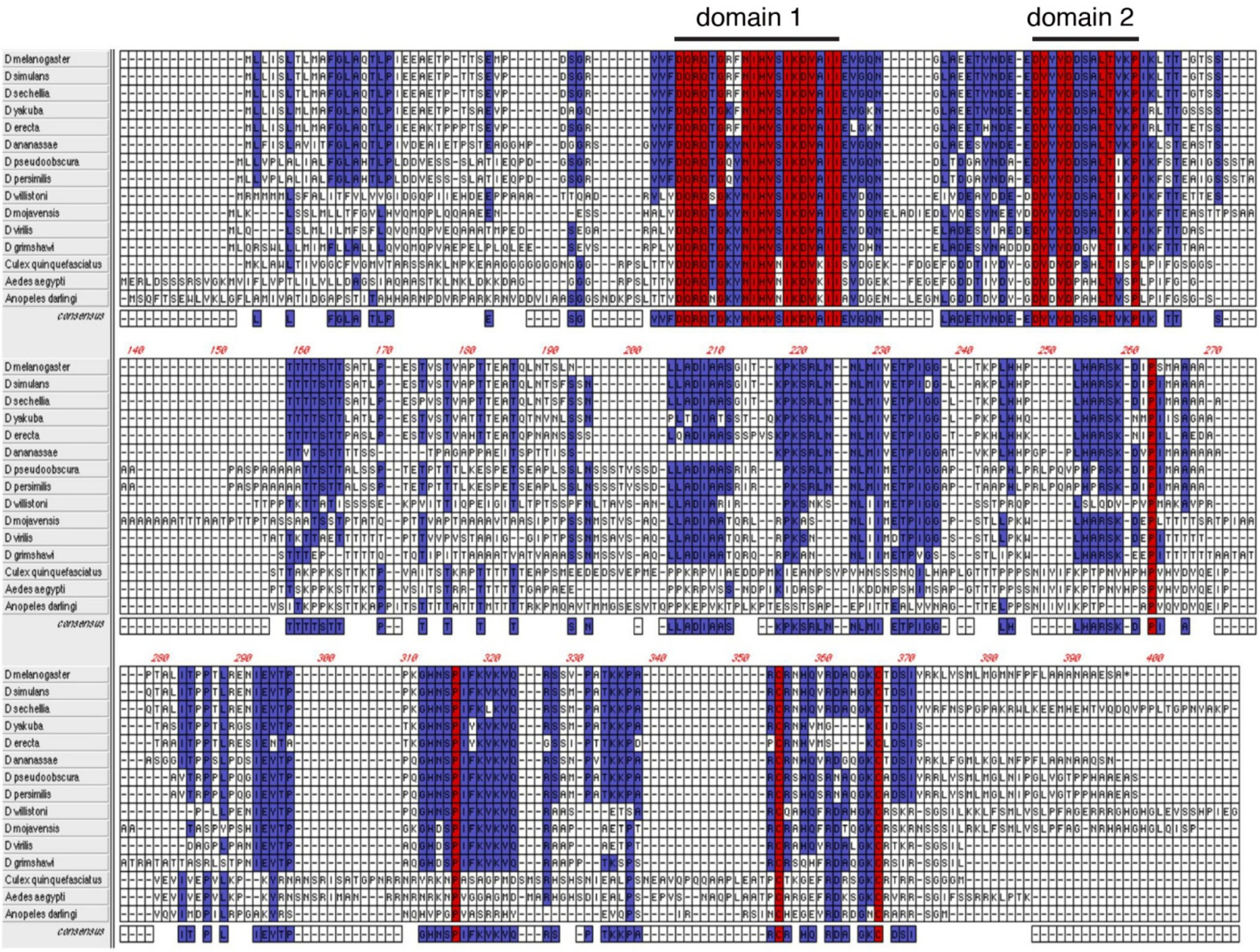

Figure S3 Hui protein alignment. The putative Hui protein of *D. melanogaster* was aligned with homologs from eleven other *Drosophila* and three other insect species. Domains of highest conservations indicated; blue indicates strong conservation; red, complete identity. The positions of the arbitrarily determined Domains 1 and 2 are indicated.
