# Supplementary material for: Microarray Comparison of Anterior and Posterior Drosophila Wing Imaginal Disc Cells Identifies Novel Wing Genes
Source: G3 (Bethesda). 2013 Aug 1;3(8):1353–62. doi: 10.1534/g3.113.006569 (PMC3737175; doi:10.1534/g3.113.006569)
Supplement: Supporting Information [file supp_g3.113.006569_TableS2.pdf]

Table S2 Protein sequences and prediction of signal peptides. The predictions for signal peptides were generated with the SignalP 4.1 software (http://www.cbs.dtu.dk/services/SignalP/) from the Center for Biological Sequence Analysis of the Technical University of Denmark Lyngby.

```
SignalP 4.0: discriminating signal peptides from transmembrane regions
Petersen TN., Brunak S., von Heijne G. & Nielsen H.
Nature Methods, 8:785-786, 2011
```

CG10200 (*hui*) predicted Protein 1

```
MKYAVIAIALFAITTASASSAGQFLPRAFFTLDSEGHQSNVHPVNAHLLR
RLRRQSSSSSSSSSSSSSSGGNVFTYASHSVQNADGSGHSGSSYTQQAAA
PVLGGVSFDERFGESSLTGGDNYYPTYNGYTSAASISSNSGVGSTGSYQH
ISGVGPIPATQIQQTVAVVDASGNQKVTNIQGATDSNGVLNIKKTESTYS
SK
```

```
# SignalP-4.1 euk predictions
```
>Sequence

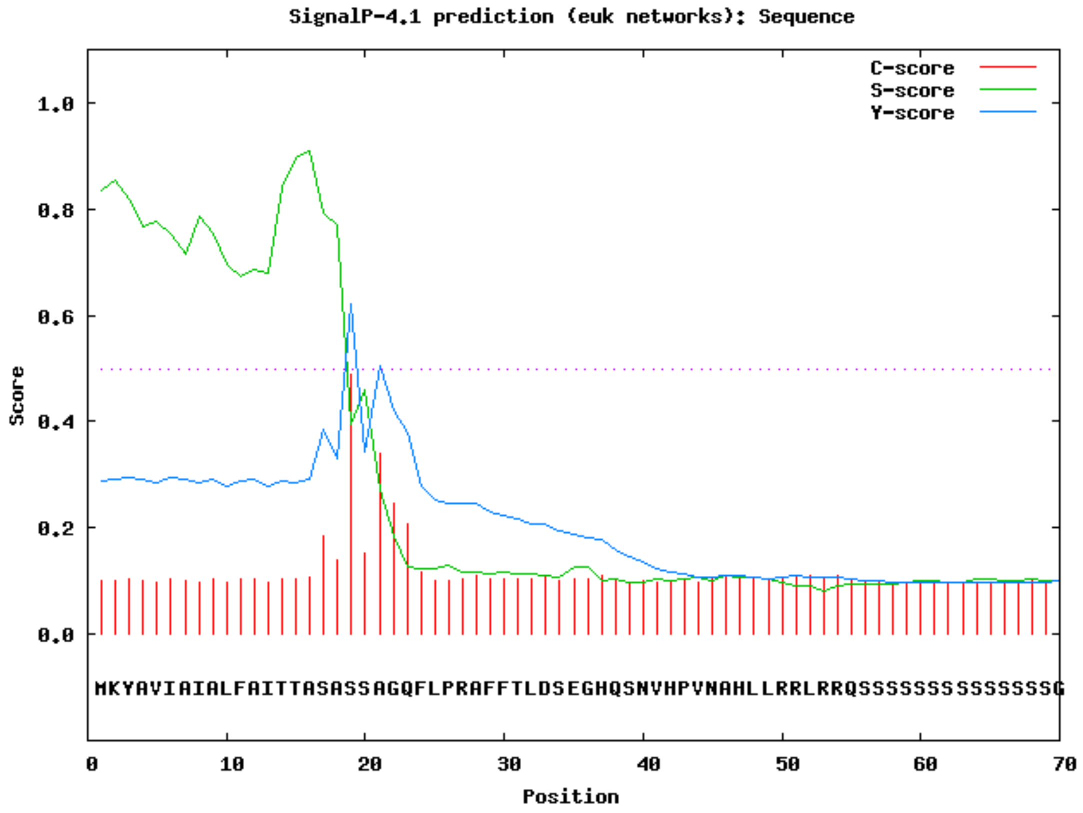

# Measure	Position	Value	Cutoff	signal peptide?
max. C	19	0.490		
max. Y	19	0.621		

```
  max. S    16       0.910

  mean S     1-18    0.778

       D     1-18    0.706   0.340   YES

Name=Sequence  SP='YES' Cleavage site between pos. 18 and 19: ASA-SS D=0.706
D-cutoff=0.340 Networks=SignalP-noTM
```

data

gnuplot script

Signal peptides: 1
processed fasta entries
gff file of processed entries

CG10200 (*hui*) predicted Protein 2

```
MKYAVIAIALFAITTASASSAGQFLPRAFFTLDSEGHQSNVHPVNAHLLR
RLRRQSSSSSSSSSSSSSSGGNVFTYASHSVQNADGSGHSGSSYTQQAAA
PVLGGVSFDERFGESSLTGGDNYYPTYNGYTSAASISSNSGVGSTGSYQH
ISGVGPIPATQIQQTVAVVDASGNQKVTNIQGATDSNGVLNIKKTESTYS
SK
```

SignalP-4.1 euk predictions
>Sequence

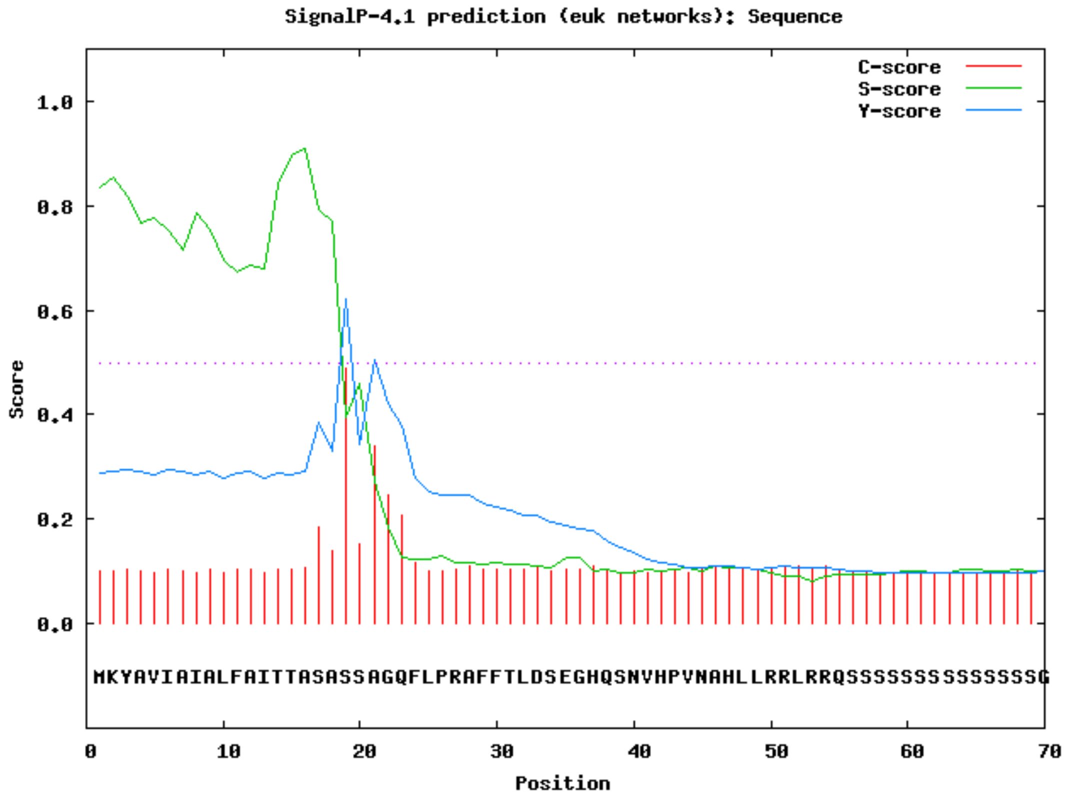

# Measure	Position	Value	Cutoff	signal peptide?
max. C	19	0.490		
max. Y	19	0.621		
max. S	16	0.910		
mean S	1-18	0.778		
D	1-18	0.706	0.340	YES

Name=Sequence SP='YES' Cleavage site between pos. 18 and 19: ASA-SS D=0.706 D-cutoff=0.340 Networks=SignalP-noTM

data

gnuplot script

Signal peptides: 1
processed fasta entries
gff file of processed entries

CG16884 predicted Protein

```
MKVFICLAALLVASACASKTEGEKVPLEKKLDKRGLLDLGYGYGHAGLDT
GYLGHGSISGHGSYGHGYGLTGYSAPAAVAVGHSGPAIAVGHTAPAVAVH
HAPAPYVISKQADVHKTITITKGIPVPVHVDRPYPVVHEKRVPVEVKVPV
PQPYEVIRKVPVTVKEYVKVPVPVPQPYEVIRHEKVPVHVPVDRPVPVEV
PRPYPVPVAKPYPVYVEKAVNVQVPVHVDRPYPVYVKVPVVSHSVVKHAP
TVAVSSYPVSAIGHDATVYSDHHGYHK
```

```
# SignalP-4.1 euk predictions
```
>Sequence

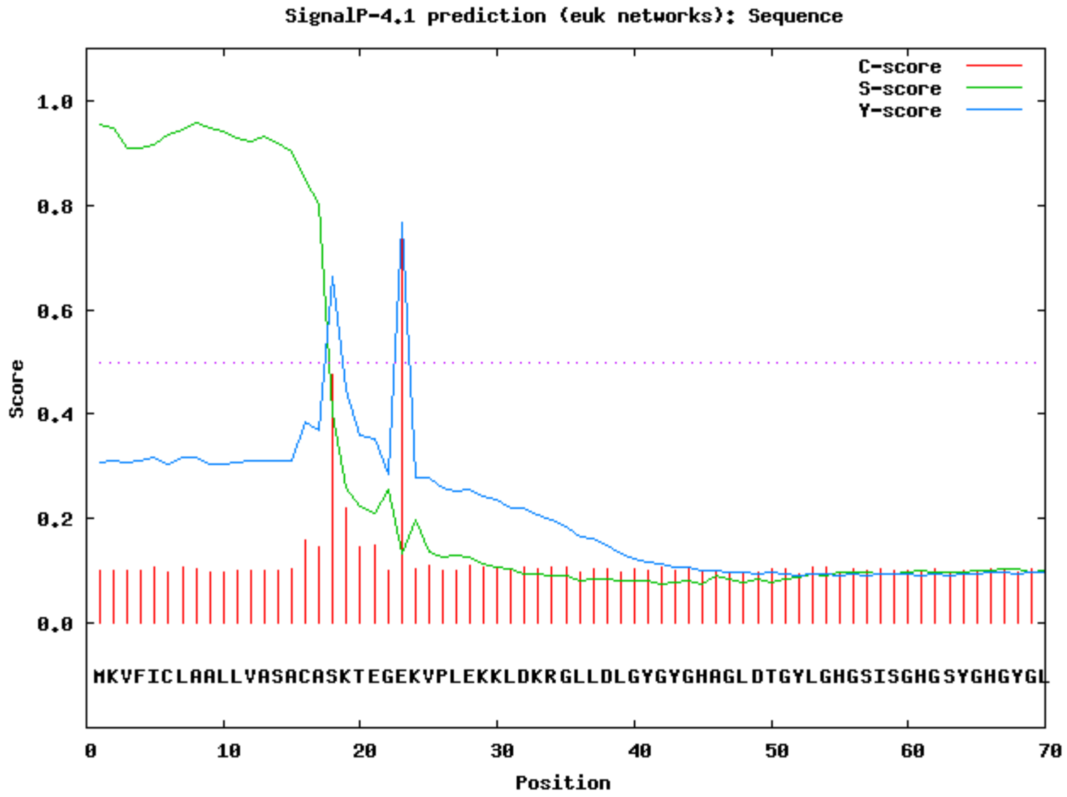

# Measure	Position	Value	Cutoff	signal peptide?
max. C	23	0.762		
max. Y	23	0.767		
max. S	8	0.958		
mean S	1-22	0.772		

D 1-22 0.770 0.340 YES

Name=Sequence SP='YES' Cleavage site between pos. 22 and 23: TEG-EK D=0.770 D-cutoff=0.340 Networks=SignalP-noTM

data

gnuplot script

Signal peptides: 1
processed fasta entries
gff file of processed entries

CG15905 predicted Protein

MLLISLTLMAFGLAQTLPIEEAETPTTSEMPDSGRVVFDQRQTGRFNIHV
SIKDVAIIEVGQNGLAEETYNDEEDYYYDDSALTVKPIKLTTGTSSTTTT
STTSATLPESTVSTVAPTTEATQLNTSLNLLADIAASGITKPKSRLNNLM
IVETPIGGLTKPLHHPLHARSKDIPSMAAAAPTALITPPTLRENIEYTPP
KGHNSPIFKVKVQRSSVPATKKPARCRNHQVRDAQGKCTDSIYRKLYSML
MGMNFPFLAAANAAESA

SignalP-4.1 euk predictions
>Sequence

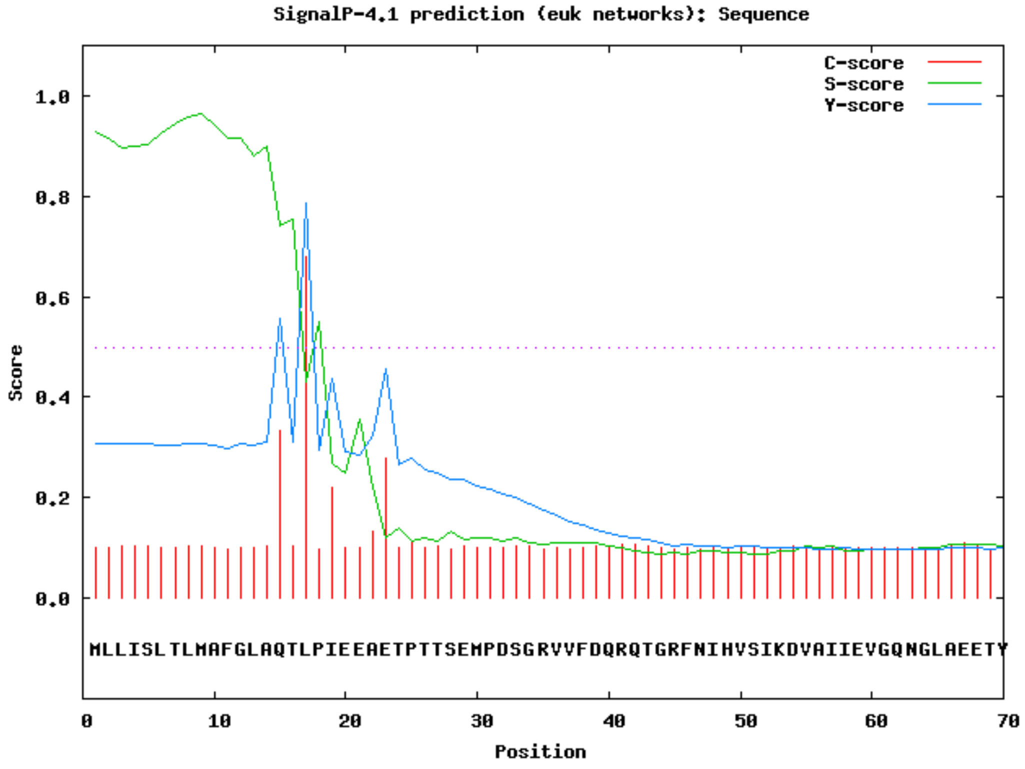

```
# Measure  Position  Value    Cutoff   signal peptide?

  max. C    17       0.680

  max. Y    17       0.785

  max. S     9       0.963

  mean S     1-16    0.899

       D     1-16    0.846   0.340   YES
```

Name=Sequence SP='YES' Cleavage site between pos. 16 and 17: AQT-LP D=0.846 D-cutoff=0.340 Networks=SignalP-noTM

data

gnuplot script

Signal peptides: 1
processed fasta entries
gff file of processed entries
